# A Regulatory miRNA–mRNA Network Is Associated with Tissue Repair Induced by Mesenchymal Stromal Cells in Acute Kidney Injury

**DOI:** 10.3389/fimmu.2016.00645

**Published:** 2017-01-03

**Authors:** Danilo Candido de Almeida, Ênio Jose Bassi, Hatylas Azevedo, Letícia Anderson, Clarice Silvia Taemi Origassa, Marcos Antônio Cenedeze, Vinicius de Andrade-Oliveira, Raphael José Ferreira Felizardo, Reinaldo Correia da Silva, Meire Ioshie Hiyane, Patricia Semedo, Marlene Antônia dos Reis, Carlos Alberto Moreira-Filho, Sergio Verjovski-Almeida, Álvaro Pacheco-Silva, Niels Olsen Saraiva Câmara

**Affiliations:** ^1^Departamento de Medicina, Divisão de Nefrologia, Universidade Federal de São Paulo, São Paulo, Brazil; ^2^Departamento de Imunologia, Instituto de Ciências Biomédicas, Universidade de São Paulo, São Paulo, Brazil; ^3^Instituto de Ciências Biológicas e da Saúde, Universidade Federal de Alagoas, Maceió, Brazil; ^4^Departamento de Pediatria, Faculdade de Medicina, Universidade de São Paulo, São Paulo, Brazil; ^5^Departamento de Bioquímica, Instituto de Química, Universidade de São Paulo, São Paulo, Brazil; ^6^Instituto Butantan, São Paulo, Brazil; ^7^Departamento de Patologia, Universidade Federal do Triângulo Mineiro, Uberaba, Brazil

**Keywords:** mesenchymal stromal cells, microvesicles, miRNAs, gene expression, cellular reprograming

## Abstract

Mesenchymal stromal cells (MSCs) orchestrate tissue repair by releasing cell-derived microvesicles (MVs), which, presumably by small RNA species, modulate global gene expression. The knowledge of miRNA/mRNA signatures linked to a reparative status may elucidate some of the molecular events associated with MSC protection. Here, we used a model of cisplatin-induced kidney injury (acute kidney injury) to assess how MSCs or MVs could restore tissue function. MSCs and MVs presented similar protective effects, which were evidenced *in vivo* and *in vitro* by modulating apoptosis, inflammation, oxidative stress, and a set of prosurvival molecules. In addition, we observed that miRNAs (i.e., miR-880, miR-141, miR-377, and miR-21) were modulated, thereby showing active participation on regenerative process. Subsequently, we identified that MSC regulates a particular miRNA subset which mRNA targets are associated with Wnt/TGF-β, fibrosis, and epithelial–mesenchymal transition signaling pathways. Our results suggest that MSCs release MVs that transcriptionally reprogram injured cells, thereby modulating a specific miRNA–mRNA network.

**Graphical Abstract:**
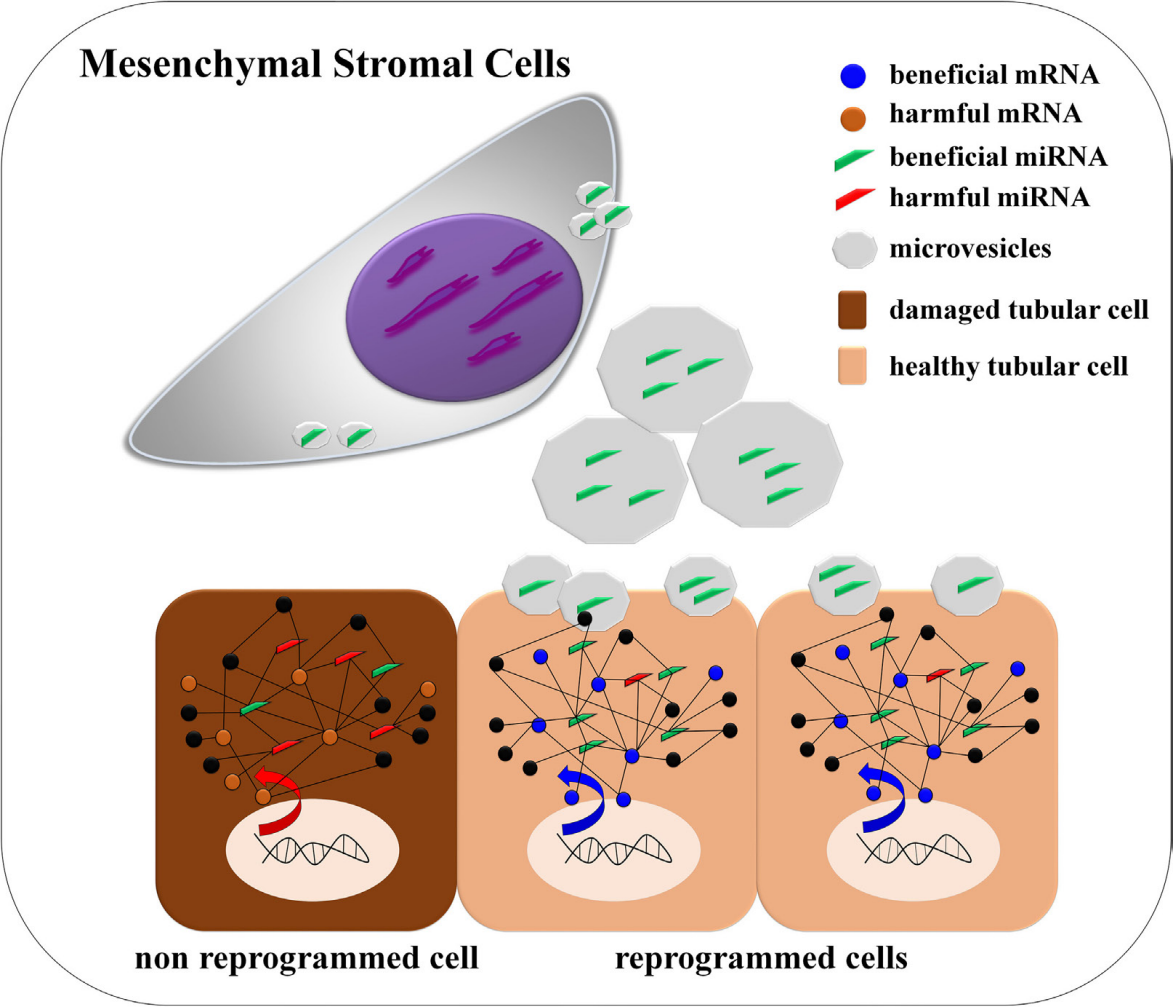
**de Almeida et al. found that mesenchymal stromal cells *via* cell-derived microvesicles modulate a miRNA/mRNA network that is linked with Wnt/TGF-β, fibrosis, and epithelial–mesenchymal transition signaling pathways. The authors detected that miRNAs miR-880, miR-141, miR-377, and miR-21 are modulated, and they can be associated to internal reprograming of tubular epithelial cells promoting tissue repair**.

## Highlights

- Mesenchymal stromal cells promote tissue cytoprotection.
- Microvesicles secreted by MSCs reprogram tubular cells.
- MSCs modulate an internal regulatory miRNA–mRNA network that is associated with tissue repair.

## Introduction

Mesenchymal stromal cells (MSCs) have a secretory potential that has been therapeutically explored in regenerative medicine using experimental models or pivotal clinical studies (1). In this context, it is known that MSCs can modulate gene expression by releasing extracellular microvesicles (MVs) to orchestrate tissue repair (2). Functionally, MSC-derived MVs (MSC-MVs) have the potential to transfer many sources of molecular information, including non-coding small regulatory RNAs, which can induce a pleiotropic effect on target cells (3, 4). Although not entirely elucidated, the mechanism of transcriptional reprograming of recipient cells seems to be achieved *via* the horizontal transfer of specific RNA species (microRNAs) that are enriched inside of MVs (5). Moreover, the MSC-MVs also contain ribonucleoproteins related to intracellular traffic and compartmentalization of RNAs (i.e., TIA, HuR, Ago2, and Stau-1 and -2), thereby highlighting the presence of an organized paracrine process that may redirect cell fate *via* cell-to-cell communication (6). In light of these properties, some evidences have suggested that the transfer of molecular information mediated by MVs plays a key role in embryonic development, tissue regeneration, pathophysiological disorders, and homeostasis dynamics (7, 8).

To date, some studies have exploited the therapeutic potential of MSC-MVs. In a model of acute kidney injury (AKI), the infusion of MSC-MVs promoted the activation of a prosurvival program in injured tubular epithelial cells with reduction of apoptosis and improvement of cell proliferation rates *via* a horizontal transfer of RNAs (9, 10). MSC-MVs have also been tested in myocardial infarction models, where they reduced the necrotic area and enhanced blood flow recovery (11, 12). Furthermore, in acute lung injury models, MSC-MV treatment reduced pulmonary edema, hypertension, and lung protein permeability and suppressed the innate inflammatory response (13, 14). Complementarily, Zhang et al. demonstrated that the injection of MSC-MVs can decrease allograft skin rejection *via* a mechanism that is dependent on Tregs polarization (15). Other studies also identified that MSC-MVs inoculation induced an efficient regeneration of sciatic nerve with the promotion of neurovascular remodeling and functional recovery after stroke (16, 17). Although these results have demonstrated the potential efficacy of MSC-MVs for tissue repair in distinct conditions, little yet is known about the precise molecular mechanisms involved in this regenerative process.

To address this issue and clarify some of the aspects related to cell-based therapies for kidney diseases, we tested here the hypothesis that MSC-MVs could promote tissue recovery by modulating a specific miRNA–mRNA regulatory network, which is able to control injury responses and chronic damage. More specifically, we used a classical toxic model of cisplatin-induced AKI and assessed how MSCs or their secreted MVs could restore renal tissue function by modulating the miRNA expression profile and a specific miRNA–mRNA network.

## Materials and Methods

### Isolation, Characterization, and Culture of MSCs and MVs

The MSCs were isolated from adult male mice inguinal adipose tissue, washed in sterile PBS (2×), minced in small pieces, and submitted to enzymatic digestion by 37°C with collagenase IA 0.1% (Sigma, USA). In sequence, these cells were filtered in cell strainer (100 µm, BD, Beckton Dickson, USA), rewashed (2×) using complete medium, and cultivated at 37°C in a humidified atmosphere with 5% CO_2_ in D-MEM low glucose culture medium (45 mM NaHCO_3_, 10% FBS, 100 U/ml penicillin, 100 U/ml streptomycin) (*n* = 5). The differentiation into mesenchymal lineages *in vitro* (i.e., adipocytes, osteoblasts, and chondrocytes) was performed using six-well plates (TPP, USA) during 15–20 days with adipogenesis, chondrogenesis, and osteogenesis Mesenchymal Stem Cell Kit (Millipore, USA), according to the manufacturer’s specifications (*n* = 5). For MV isolation, the 24h MSC serum-free supernatant was submitted to pre-centrifugation at 2,000 *g* for 5 min and after to ultracentrifugation at 100,000 *g* for 2 h. Then, the pellet was suspended in sterile PBS with 0.1% of BSA and kept at −80°C. Furthermore, MVs were evaluated according to their size properties using a NanoSight instrument (Malvern, UK). The content of small RNAs or miRNAs inside of MVs was verified with a capillary electrophoresis method using the Agilent small RNA kit, strictly following the manufacturer’s instructions (Agilent Technologies, Santa Clara, CA, USA) (*n* = 5). Two additional groups consisting of MVs treated with RNAse A (100 µg/mL, Thermo Scientific, USA) and ultracentrifuged medium without cells were carried out concomitantly, and all samples were read with an Agilent 2100 Bioanalyzer (Agilent Technologies, Santa Clara, CA, USA) (*n* = 5). The immunophenotyping of MSCs and MVs was performed using specific sets of antibodies (i.e., CD44, CD90, CD105, CD73, CD45, CD34, CD11c, CD106, CD31, CD9, and CD69, BD Bioscience, USA), according to the manufacturer’s recommendations (*n* = 5). Briefly, cells were washed (PBS), centrifugated (2,000 *g* at 5 min), and incubated (45 min in dark room) with specific antibody at 1:100 dilution and rewashed afterward in FACs buffer (PBS 2% FBS). A FACSCanto II flow cytometer (BD, Beckton Dickson) was used for cell acquisition, and the FlowJo software was used for data analysis.

### Coculture of MSCs or MVs with Renal Tubular Cells

For *in vitro* assays, approximately 2 × 10^5^ of renal epithelial tubular cells (MM55.K, ATCC^®^ CRL-6436TM) were seeded in six-well plates (TPP, USA) and treated with nephrotoxic drug cisplatin (8 µg/mL) for 48 h, and two additional treated groups with cisplatin were co-cultured in contact with MSCs (v/v 1:1, 1 × 10^5^) or MVs (50 µg/ml, sequentially each 6 h) for 48 h (Figure S4C in Supplementary Material). Subsequently, cells were trypsinized and subjected to analysis for apoptosis, cell proliferation, and oxidative stress analysis using the respective kits: Alexa Fluor^®^ 488 annexin V/Dead Cell Apoptosis kit, CellTrace™ Violet Cell Proliferation kit, and MitoSOX™ Red Mitochondrial Superoxide Indicator kit (Life Technologies, USA), following the manufacturer’s recommendations (*n* = 6). For apoptosis analysis, a range of cisplatin was utilized as death curve (1–10 µg/mL) and newly a dose of 8 µg/mL was established for additional assays (Figure S4D in Supplementary Material). Two additional groups without co-culture were processed with tubular cells treated and untreated with cisplatin. During the flow cytometry analysis to distinguish the tubular cells population (MM55.K cells) from MSCs, the MSCs population were previously labeled with the fluorescent dye CellTrace™ Violet (Cell Trace Violet Proliferation Kit, Life Technologies, USA) and excluded from analysis using negative gate strategy in flow cytometry workflow (Figure S4E in Supplementary Material). For visualizing the MVs incorporation process, the MVs were prelabeled with PKH26 red-fluorescent dye (Sigma, USA) and co-cultured in chamber slide system with tubular cells (1 × 10^4^/well) treated with cisplatin (8 µg/mL) in a time-course assay per 24 h. Images were recorded at each time point to observe MVs incorporation into tubular cells (NIS elements microscope Nikon).

### Animal Experiments and Study Groups

For all animal experiments, we used adult C57BL/6 mice, aged 8–12 weeks with 20–30 g weight (*n* = 8 *per* group). The animals were housed in polypropylene boxes at controlled room temperature and light conditions (22°C and 12-h light/12-h dark). They received a solid diet and filtered water *ad libitum*. All animal handling protocols were approved by the local Animal Care committee CEP/UNIFESP No. 1058/10. Animals were separated into three study groups according to the experimental procedures: mice treated with saline solution, mice treated with cisplatin, and mice treated with cisplatin but also transplanted with MSCs or MVs.

### *In Vivo* Model of AKI and Infusion of MSCs or MVs

To develop the *in vivo* experimental model of toxic AKI, an intraperitoneal infusion with 15 mg/kg of cisplatin (Pfizer, USA) was performed (*n* = 8 *per* group). The control group was administered with 300 µL phosphate buffer (PBS). After 24 h of cisplatin treatment (day + 1), 1 × 10^6^ of MSCs or 300 µL of saline solution (Sham group) were infused intraperitoneally in the animals. Conforming pre-established by our lab (data not showed) and in classical works in the literature (6, 10), around 100 µg of MVs suspension was infused intravenously on day +1 and day +3 after cisplatin infusion (Figure S4B in Supplementary Material). To evaluate the action mechanism of MVs during *in vivo* infusion, three additional groups were set in parallel: (i) MVs pretreated with Proteinase K 20 µg/mL (Sigma, USA) and DNase I (10 U/µL; Stratagene, USA); (ii) MVs pretreated with a cocktail of proteases [Proteinase K 20 µg/mL (Sigma, USA), trypsin (Life Technologies, USA) and collagenase 1 A (Sigma, USA)]; and (iii) MVs pretreated with Proteinase K 20 µg/mL (Sigma, USA) and RNase A (100 mg/mL; Thermo Scientific, USA), according to the manufacturer’s instructions. At day 4 after cisplatin administration, with or without MSC or MV infusion, all groups were euthanized using an overdose of anesthetic [xylazine (30 mg/kg) + ketamine (200 mg/kg)], and urine, blood, and renal tissues were collected and maintained at −80°C until analyses.

### Renal Physiological Parameters

The creatinine, urea, and lactate analysis were performed to measure the status of renal function (*n* = 8). Creatinine was measured using the Jaffe modified method (Labtest Diagnostic, Brazil). Urea concentration was analyzed with a urea kit (Labtest Diagnostic, Brazil), according to the manufacturer’s specifications. The percentage of lactate was measured using Advia 1650 equipment (Bayer, Germany), following the datasheet instructions. All animals were periodically (each 24 h during 4 days) monitored for gain or loss of body mass (*n* = 8). Finally, an injury score evaluation was determined according to the absolute mean of clinical parameters (Table S3 in Supplementary Material).

### Histomorphometry of Kidney Tissue

Kidney fragments were fixed in buffered formaldehyde 4% (pH = 7.0) and embedded in paraffin block. For each group, histological sections of 4–5 µm were performed and the slides were stained with hematoxylin/eosin (H&E). For a quantitative evaluation of renal injury, clinical pathologic score of tissue damage was performed. Twenty-five fields from five slides were selected (in magnification 400×), and for all groups, the percentage of necrosis, expansion of glomerular cavity, tubular dilation, detachment of epithelial cells, formation of renal casts, and inflammatory infiltrates were evaluated. All these parameters were determined here as tissue injury index. A pathological score was assigned for each parameter, according to injury severity (0 = absent, 1 = mild, 2 = moderate, and 3 = severe). Finally, an overall percentage score (e.g., arithmetic mean of all absolute parameters) was generated following a direct comparison between the experimental groups.

### *In Situ* Immunostaining

Tissue sections of 3–4 µm were processed on silanized slides (*n* = 5). The immunohistochemistry was performed using protein block reagent, an EnVision+/HRP-Dual Link kit, DAB (3-3) tetrahydrochloride and diaminobenxidine solution (Dako, Denmark), and caspase 3 antibodies (1:1,000, Cell Signaling, USA), following the manufacturer’s specifications. Slides were hydrated and dehydrated through alcohol–xylene gradient (30–100%), washed in buffer, and stained and mounted in Permount solution (Sigma, USA). To check the percentage of extracellular matrix deposition, picrosirius staining (Sirius red in picric acid 0.1%) was performed. For immunofluorescence evaluation, kidney tissue fragments were frozen in Tissue Tek (Sakura, Japan) and 4-µm cryosections were processed. Cells were fixed, permeabilized, and incubated with primary antibodies (1:200), i.e., anti-Ki-67 (Abcam, USA), followed by secondary antibodies (1:400) conjugated to FITC (Abcam, USA). Cells were also counter-stained with DAPI for nuclei visualization (Vector Labs, USA). The fluorescent labeling was observed using fluorescence microscopy (Nikon, Japan). For the detection of *in situ* necrosis/apoptosis, the TUNEL assay was used with the *in situ* Cell Death Detection Kit (Roche, Germany), following the general information contained in the manufacturer’s protocol. The following *in situ* assays such as (i) renal fibrosis by picrosirius method, (ii) cell proliferation (immunohistochemistry to Ki-67), and (iii) cell death (immunohistochemistry to Caspase-3) were performed using the quantification of at least 20–30 fields (magnification 200×) with software developed by Nikon (NIS elements microscope Nikon).

### Assessment of Oxidative Stress and Determination of Cytokines

The *in vivo* tissue oxidative stress was evaluated indirectly by nitric oxide (NO) measurement using the Griess modified method (*n* = 6). In addition, the renal content of reduced (GSH) and oxidized (GSSG) glutathione levels were assessed using a glutathione fluorescent detection kit (BioVision, USA), following the manufacturer’s instructions (*n* = 6). The animal serum cytokine profile was performed using a flow cytometry beads array systems with Cytometric Bead Array Kit for inflammation (BD Bioscience, USA), according to the manufacturer’s recommendations (*n* = 6).

### miRNA Expression Profiling

Total RNA, including small RNAs (miRNAs), was extracted from all kidney tissues with a miRNeasy Mini Kit (50) (Qiagen, South Korea), according to the manufacturer’s instructions (*n* = 6 *per* group). The concentration, quality, and integrity of RNA samples were, respectively, checked using a Nanodrop spectrophotometer (Thermo Scientific, USA) and Bioanalyzer equipment (Agilent Technologies, USA) (*n* = 6 *per* group). Furthermore, the reverse transcription of total RNAs and miRNAs was performed using, respectively, the High Capacity kit (Life Technologies, USA) and the RT^2^ miRNA First Strand Kit (12) (Qiagen, South Korea) (*n* = 6 *per* group). Gene expression levels between the groups of samples were measured by real-time PCR (RT-qPCR) using the TaqMan system (Life Technologies, USA) (*n* = 6). To translate miRNAs into cDNAs was used the miScript II RT Kit, following all recommendations in datasheet (Qiagen, South Korea). For quantitative detection of pre- and mature miRNAs forms, the Syber Green PCR kit was utilized (Qiagen, South Korea). At gene expression assays normalization, the endogenous HPRT gene was used as a housekeeping gene, whereas that for miRNA reactions, the endogenous control SNOR73A was used. Finally, the reaction was carried out using the ABI Prism 7300 sequence detection system (Life Technologies, USA). The results were analyzed through relative quantification method according to formula: 10,000/2^ΔCt^, using SDS software (Life Technologies, USA). The global miRNA PCR arrays were performed in 96-well plates per each set (3 set *per* group), following the recommendations specified in the products catalogs: RT^2^ SYBR Green ROX qPCR Master Mix (24) and Mouse miRNome RT^2^miRNA PCR array (528 miRNAs; Qiagen, South Korea). Data analysis and normalization were performed using the web-based application provided on the manufacturer’s website (Qiagen, South Korea).

### Network Analysis of Differentially Expressed miRNAs and Their mRNA Targets

Differentially expressed miRNAs were selected between the groups using the QIAGEN software website analysis. Only the miRNAs that had at least fivefold changes in expression were included in further analysis. Furthermore, the MIR@NT@N resource was used to identify mRNAs that were targeted by the selected differentially expressed miRNAs (18). Then, we searched for overlaps in the miRNA sets using GeneVenn, a web application for comparing set lists using Venn diagrams (19). The known and predicted relationships between miRNAs and target genes were obtained from the DIANA Tools and MIR@NT@N resource, according to the criteria in the default parameters (18–21). We only selected those genes targeted by at least two miRNAs to restrict our analysis to the most relevant pairs of connected miRNAs and mRNAs. Pathways significantly enriched with target genes were identified with the DIANA Tools. We also generated miRNA–mRNA networks to visualize the relations between miRNA and their predicted target genes. The gene ontology (GO) analysis were generated using a web-based enrichment software analysis, FunNet, and the networks were built using the Cytoscape software, which allows network editing and visualization of the molecular interactions (22).

### Overexpression and Silencing of Selected miRNAs

To validate our findings, cultures of renal tubular cells (2 × 10^5^ cells per well) were seeded in six-well plates to further addition of RNA oligos, in order to inhibit or to overexpress specific miRNAs (i.e., miR-21, miR-377, miR-880, and miR-141). The cells were submitted to a transfection process using the N-TER kit (Sigma, USA), conforming to the manufacturer’s instructions (Qiagen, South Korea) (*n* = 6). According at its respective oligos, the cells were transfected with miRNAs mimics, inhibitors or scramble oligos. Additionally, a control group was run in parallel with transfection reagent alone. Subsequently, all groups were submitted to cisplatin for 48 h, and MV treatment was performed in an additional group conforming previously described.

### Western Blotting

For each sample, 50 µg of renal tissue protein extract was used for electrophoresis on 10% polyacrylamide gels (SDS-PAGE). The immunostaining was carried out with primary antibodies (β-actin/1:1,000, Sigma, USA; IKK-α/1:1,000, Cell Signaling, USA; Argonaute 2/1:1,000, Cell Signaling, USA; Drosha/1:1,000, Cell Signaling, USA; Dicer/1:1,000, Imgenex, India), followed by conjugated secondary antibodies (anti-mouse or anti-rabbit peroxidase/1:5,000, Sigma, USA). Then, the membrane was revealed by chemiluminescence methods using the ECL kit (Millipore, USA), and the images were acquired on GEN-BOX equipment (Syngene, UK) (*n* = 5). The GeneSnap software and GeneTools (Syngene, UK) were used to identify, analyze, and quantify the gel bands.

### Statistical Analysis

Data were collected and presented according to classical descriptive statistics. The data sets were tested for a Gaussian distribution by a Kolmogorov–Smirnov test with Dallal–Wilkison–Lillie for a *p* value, and further statistical inference parametric methods were performed for all analyses. Student’s *t* and one-way ANOVA tests (followed by Tukey post-test) were used to determine significant differences, respectively, between two or more independent sample groups. All experimental data obtained in this study are represented by the mean and SD of the mean (±SD), and statistical analyses were conducted considering a minimal statistical significance (α) at 5% (*p* < 0.05).

## Results

### MSC and MSC-MV Treatments Promote Functional Recovery

**Figure 1:**
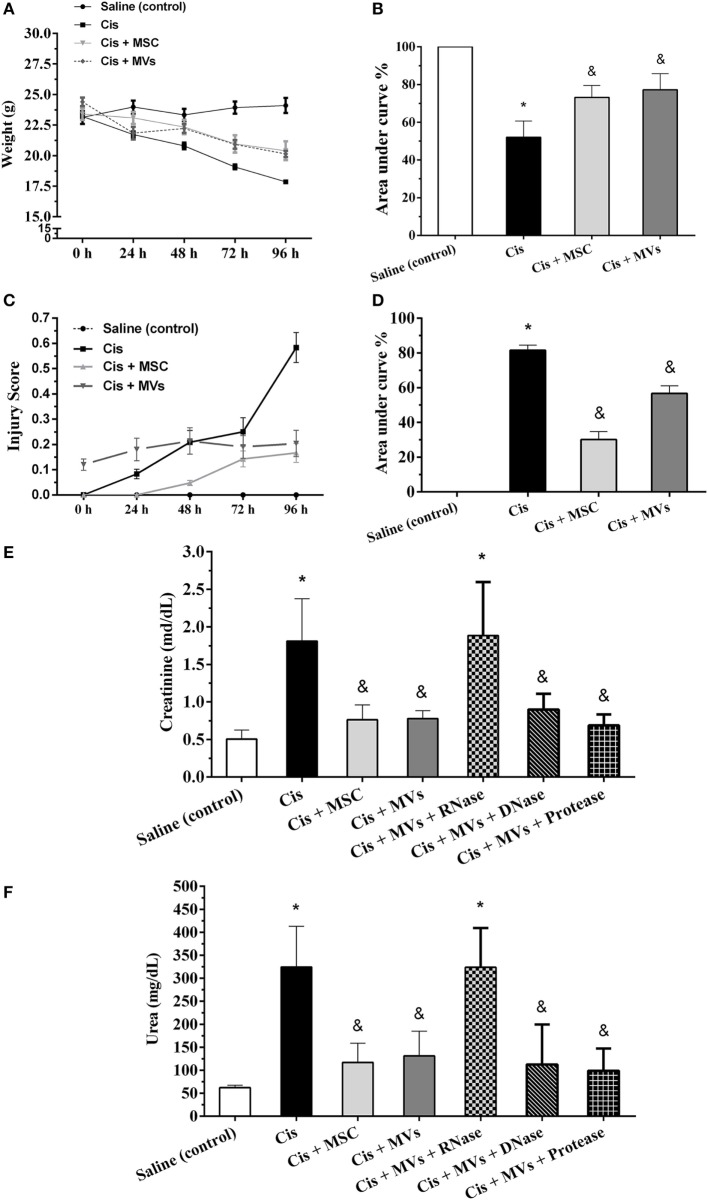
**MSC and MV treatments promote tissue recovery**. **(A,B)** Evaluation of weight loss; **(C,D)** determination of injury score; **(E)** serum creatinine levels; and **(F)** serum urea levels (*p < 0.05 compared with the saline/control group and ^&^*p* < 0.05 compared with the cisplatin group). Cis, cisplatin; MSC, mesenchymal stromal cell; MVs, microvesicles.

First, we performed an extensive characterization of MSCs and their MVs. MSCs *in vitro* showed the classical features of mesenchymal cells such as fibroblast colony-forming unit formation, fibroblast-like morphology, differentiation into mesodermal lineages (i.e., adipocytes, chondrocytes, and osteoblasts), high proliferation index, and a distinctive immunophenotype with classical MSCs features (Figure S1 in Supplementary Material). On the other hand, MVs secreted by MSCs displayed spheroid morphology, had small size (≈125 nm), exhibited positivity to MSC (i.e., CD105, CD90, and CD44), exosomes (i.e., CD9 and CD63), and surface markers, and internally had the presence of miRNAs (<22 nt) and their biogenesis machinery (Dicer, Drosha, and Argonaut 2) (Figure S2 in Supplementary Material). Subsequently, we assessed the repair potential of MSCs and MSC-MV treatment in a toxic model of AKI. Interestingly, we demonstrated *in vivo* after cisplatin administration that injections of both MSCs and MVs ameliorated physiologic injury parameters such as weight loss, injury scores, and renal damage markers (serum creatinine and urea levels; Figures 1A–F; Movies S1 and S2 in Supplementary Material). Moreover, to understand which molecular pattern inside MVs was promoting tissue repair, we performed a pretreatment of MVs with RNAses and observed the abrogation of the therapeutic effect. By contrast, DNAses and proteases pretreatments did not show any effect (Figures 1E,F). Furthermore, we identified *in situ* in histological renal sections treated with MSCs and MVs an improvement in tissue regeneration status as observed by increase in the proliferation rate (Ki-67 staining) and reduction of the injury score, detected mainly by reduction of apoptosis index (active caspase 3 and TUNEL assays) and interstitial extracellular matrix deposition (Sirius red staining) (Figure S3 in Supplementary Material).

### MSC and MSC-MV Therapies Modulate a Set of Prosurvival Molecules

**Figure 2:**
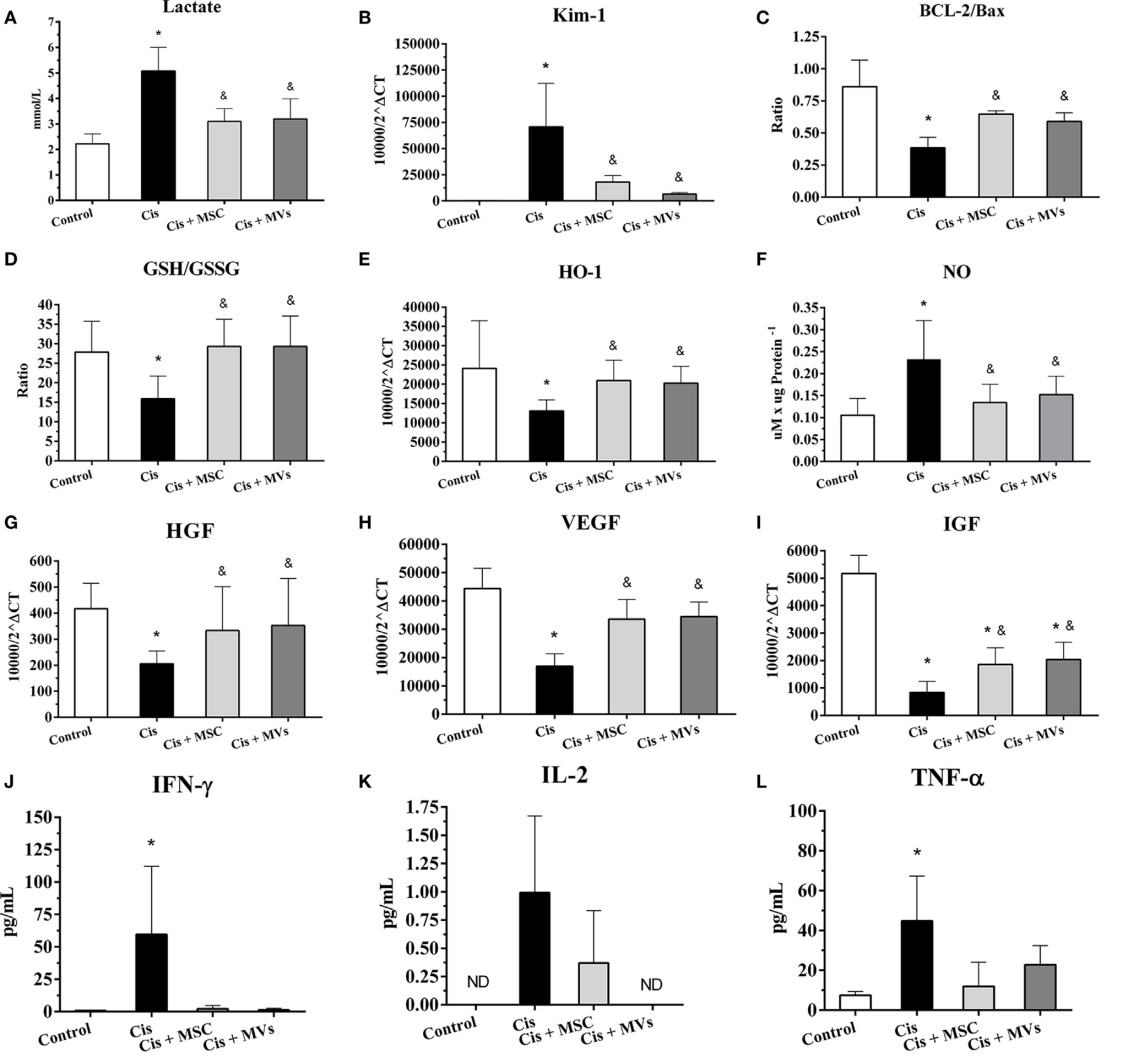

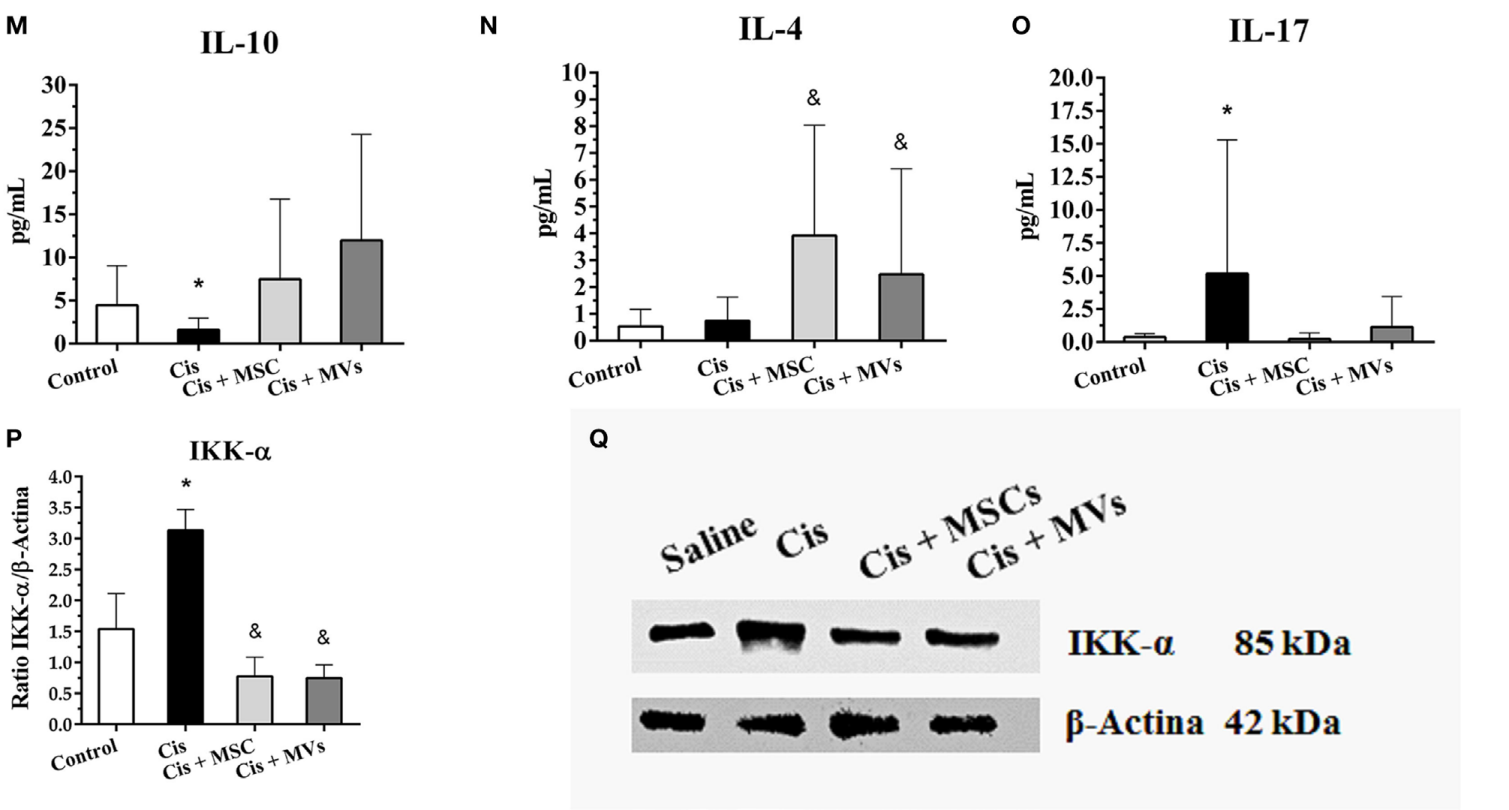
**Injury- and protective-related molecules are modulated after MSC or MV treatments**. **(A)** Serum lactate levels; **(B)** Kim-1 expression, a renal damage marker; **(C)** BCL-2/Bax ratio; **(D)** glutathione reduced (GSH) and oxidized (GSSG) ratio (GSH/GSSG); **(E)** expression of heme oxygenase 1 (HO-1); **(F)** tissue levels of nitric oxide (NO); **(G)** expression of hepatocyte growth factor; **(H)** expression of vascular endothelial growth factor; **(I)** expression of insulin-like growth factor type 1 (IGF); **(J)** serum levels of interferon gamma (IFN-γ); **(K)** serum levels of interleukin 2; **(L)** serum levels of tumor necrosis factor α (TNF-α); **(M)** serum levels of interleukin 10; **(N)** serum levels of interleukin 4; **(O)** serum levels of interleukin 17, and **(P,Q)** protein expression of IKK-α kinase (*p < 0.05 compared with the saline/control group and ^&^*p* < 0.05 compared with the cisplatin group). Cis, cisplatin; MSC, mesenchymal stromal cell; MVs, microvesicles.

In an attempt to investigate the beneficial therapeutic effect of MSCs, we analyzed *in vivo* the tissue expression of molecules that are involved in the modulation of renal toxic injury. With this analysis, we detected in kidney tissues after MSC and MV therapy a reduction of the injury markers (e.g., lactate index and Kim-1 levels) and apoptosis index (i.e., Bcl-2/Bax ratio) when compared to damage group alone (cisplatin) (Figures 2A–C). Moreover, the MSC and MV infusion decreased the renal oxidative stress by enhancing anti-oxidant enzymes levels (e.g., GSH/GSSG ratio and HO-1) and reducing free radicals presence (i.e., NO; Figures 2D–F). Concomitantly, we observed in kidney tissues treated with both MSCs and MVs an increase in the expression of classical cytoprotective genes such as hepatocyte growth factor, vascular endothelial growth factor, and IGF (Figures 2G–I). In addition, mice transplanted with MSCs or MVs showed a decrease in the serum inflammatory cytokine levels (i.e., interleukin 2, interleukin 17, TNF-α, and INF-γ) and an increase in anti-inflammatory molecules such as interleukin 10 and interleukin 4 (Figures 2J–O). Finally, the NF-κB signaling pathway, which is associated with acute inflammation, was also downregulated in renal tissues after MSCs and MVs intervention, as demonstrated by the reduction of IKK-α protein expression (Figures 2P,Q).

### MSC and MSC-MV Treatments Promote *In Vitro* Cytoprotection and Cellular Recovery

**Figure 3:**
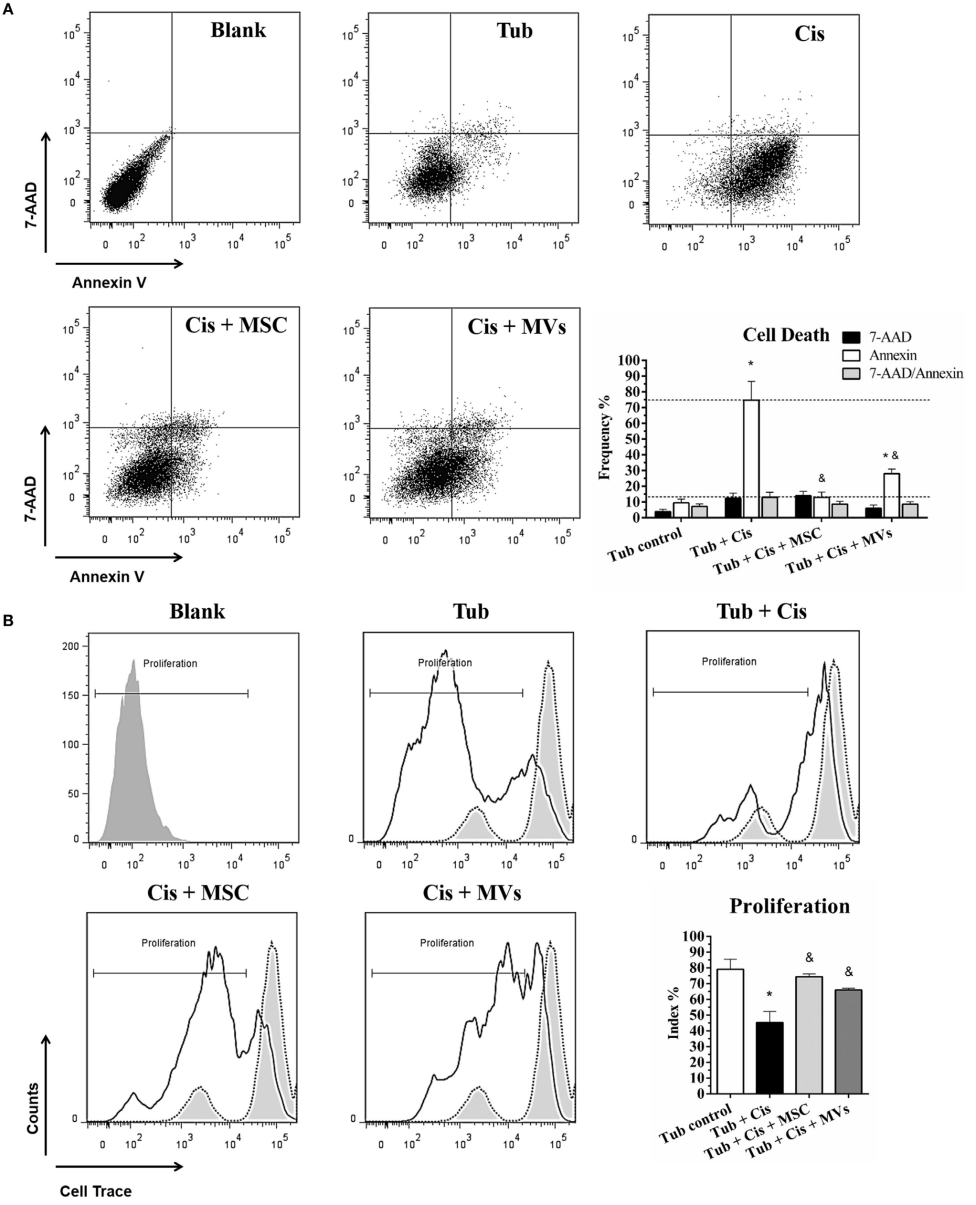

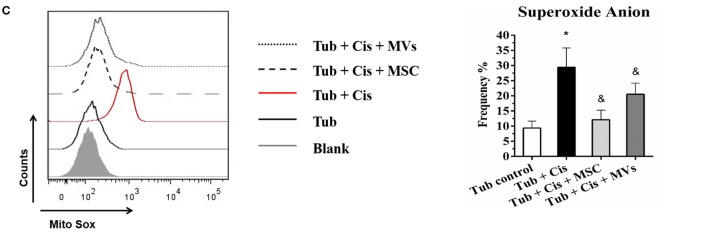
**MSC and MV treatment promoted cell recovery (A)** Evaluation of cell death, apoptosis (Annexin V), and the necrosis (7-AAD) index; **(B)** determination of cell proliferation score (Cell Trace Ratio); and **(C)** analysis of oxidative stress and superoxide anion radical activity ${(\text{O}}_{2}^{\cdot -})$ (*p < 0.05 compared with the saline/control group and ^&^*p* < 0.05 compared with the cisplatin group). Cis, cisplatin; MSC, mesenchymal stromal cell; MVs, microvesicles; Tub, murine renal tubular epithelial cell line MM55.K.

To support our *in vivo* findings, we evaluated the cross-talking between MSCs and/or MVs with tubular epithelial cells. Using *in vitro* co-culture assays, we observed after cisplatin stimulus that both treatments (MSC and MVs) reduced cell death frequency (i.e., apoptosis/necrosis ratio) and oxidative stress index (i.e., anion superoxide level) while promoting an increase in the cellular proliferative rates, thereby indicating that MSCs and MVs can restore the tubular epithelial cell physiology after toxic injury (Figure 3).

### MSC Treatment Modulates the Global miRNA Profile in Renal Tissue

**Figure 4:**
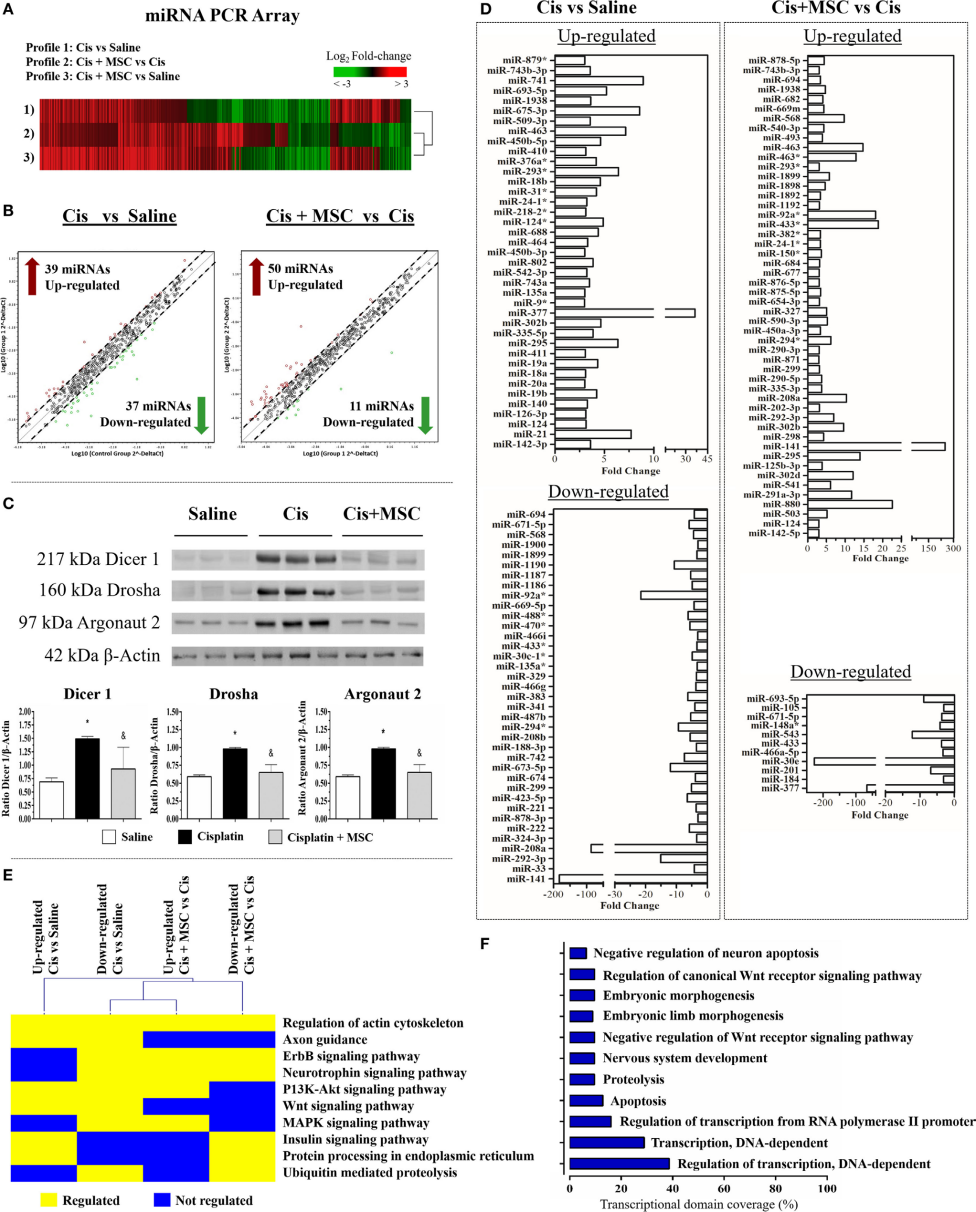
**The global miRNA signature of renal tissues after MSC treatment**. **(A)** Heat map analysis of miRNA profile for all comparisons; **(B)** scatter plots representing the differentially expressed miRNAs with a fold change ≥3: (i) cisplatin in comparison to saline and (ii) cisplatin + MSC in comparison with cisplatin alone; **(C)** expression levels of proteins involved in miRNA biogenesis (Dicer 1, Drosha, and Argonaut 2); **(D)** bar plot of differentially expressed miRNAs with a fold change ≥3 at each comparison; **(E)** signaling pathways associated to genes predicted to be target of the top differentially expressed miRNAs; and **(F)** gene ontology analysis of biological processes linked to the top regulated miRNAs for all comparisons (*p < 0.05 compared with the saline/control group and ^&^*p* < 0.05 compared with the cisplatin group). Cis, cisplatin; MSC, mesenchymal stromal cell; MVs, microvesicles.

In search for precise molecular mechanisms involved in the MSC-mediated cytoprotection, we analyzed the global miRNA profile changes in the renal tissue submitted to toxic injury alone or treated with MSCs (Figure 4A). We detected a total of 528 miRNAs expressed in common between all the tested conditions (Figure 4A; Figure S1 in Supplementary Material), in which 39 were upregulated and 37 were downregulated in the cisplatin condition when compared with saline at a fold change ≥3 (Figures 4B,D). After MSC therapy, 50 miRNAs were upregulated and 11 miRNAs were downregulated in the cisplatin + MSCs condition when compared with cisplatin alone, considering a fold change ≥3 (Figure 4B). Further, we observed that the proteins related to miRNA biogenesis (e.g., Dicer, Drosha, and Argonaut 2) were all modulated during toxic injury, and after MSC administration, they had their physiologic levels re-established (Figure 4C). These results were complemented by miRNA amount analysis, which showed higher miRNA levels during injury process and lower levels after MSC treatment (Figure S4A in Supplementary Material). Additionally, we observed that the differential changes in the miRNA signatures were associated with a broad modulation of specific miRNAs associated with cisplatin or MSC intervention (Figures 4C,D). Therefore, we decided to narrow our downstream analysis to the highly modulated miRNAs (fold change ≥5) at each comparison. In the cisplatin condition compared to saline, we found that miR-377, miR-741, miR-675-3p, miR-21, miR-463, and miR-293* were the most upregulated miRNAs, whereas miR-141, miR208a, miR-92a*, miR-292-3p, miR-673-5p, and miR-1190 were the most downregulated miRNAs (Figure 4D). When we evaluated the cisplatin + MSCs condition in comparison with cisplatin alone, we detected the miR-141, miR-880, miR-433, miR-92a, miR-463, and miR-295 as most upregulated miRNAs, while the miR-30e, miR-377, miR543, miR-693-5p, miR-201, and miR148a* were the most downregulated ones (Figure 4D). Subsequently, we identified the protein-coding genes that were predicted to be targets of the top differentially expressed miRNAs at each comparison and looked for biological pathways that were significantly enriched with those genes. Thus, with this approach, we found signaling pathways related to tissue repair and homeostasis recovery, such as regulation of actin, axon guidance, ErbB, P13K-Akt, Wnt, MAPK, insulin, protein processing, and ubiquitin/proteolysis (Figure 4E). A brief description of all pathways associated with the differentially expressed miRNAs at each comparison and their predicted target genes is illustrated in Table S2 in Supplementary Material. In addition, the GO analysis using the mRNAs targeted by miRNAs showed that the majority of the highly regulated miRNAs in renal tissues are linked to positive regulation of transcription DNA-dependent biological processes, thereby highlighting a presence of intrinsic regulatory mechanism (Figure 4F). In attempt to verify if the miRNAs differentially expressed in renal tissues were directly transferred by MVs, we carried out a miRNAs profile of MVs and MSCs and detected a higher number of miRNAs upregulated in MVs than MSCs (Figure S5A in Supplementary Material). Moreover, we identified that the top modulated miRNAs were not directly associated to kidney miRNA profile and the miR-377 and miR-141 (mostly regulated in renal tissues), although present inside of MVs, they were not found to be enriched into MVs (Figures S5B,C in Supplementary Material). These findings suggest that MVs and MSCs did not share the same miRNA profile and its effect in the renal tissue can be conducted by an indirect regulatory process.

### An Integrative Network Analysis Revealed the Role of Specific miRNAs in AKI and in Cytoprotection Promoted by MSCs

**Figure 5:**
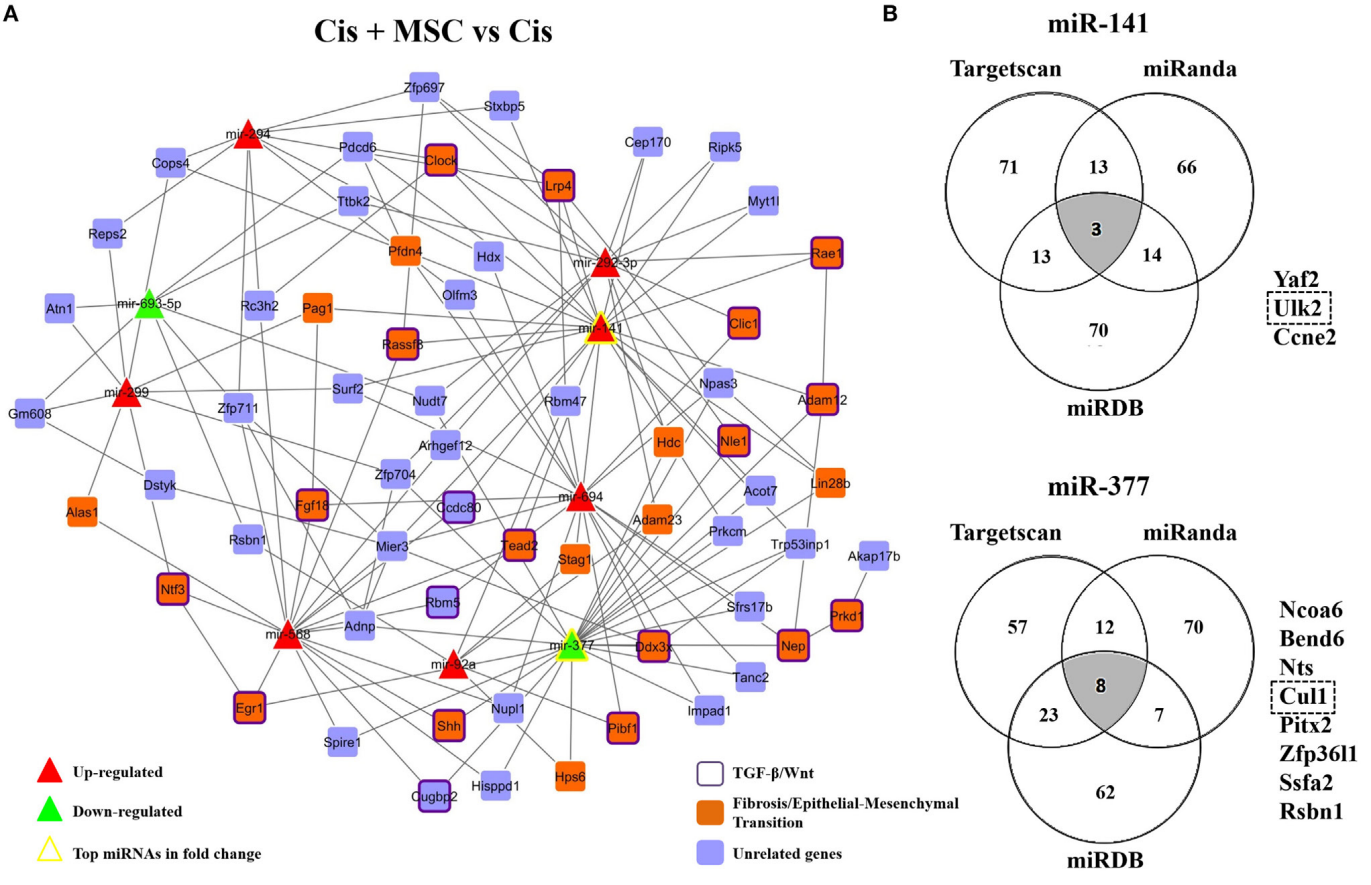
**miRNA–mRNA network in cytoprotection induced by MSCs**. **(A)** MSCs-modulated network showing the interactions between miRNAs modulated by MSCs and putative targets genes interacting with at least two of these altered miRNAs, and **(B)** Venn diagrams depicting overlapping miRNA target genes obtained from three microRNA target prediction tools (TargetScan, miRanda, and miRDB) and a list of overlapping potential target genes for the top two differentially expressed miRNAs in the MSCs-modulated network (i.e., miR-141 and miR-377). Upregulated and downregulated miRNAs are represented, respectively, as red and green triangles. The top miRNAs in terms of fold changes had their border colors altered to yellow. Genes previously linked to fibrosis or epithelial–mesenchymal transition are represented as orange rectangles. Genes that were previously related to the TGF-β or Wnt pathways additionally had their border colors altered to purple and unrelated genes are represented in blue rectangles. Cis, cisplatin; MSCs, mesenchymal stromal cells; MVs, microvesicles.

In order to identify some biological functions attributed to MSC renoprotection, we carried out an *in silico* analysis with the most upregulated and downregulated miRNAs in the cisplatin condition compared to saline and connected these miRNAs with previously described differentially expressed genes which were associated to cisplatin damage in renal tissues (Figure S6A in Supplementary Material) (23, 24). After cisplatin intervention, we observed that the upregulated and downregulated miRNAs (red/green triangles) in renal tissues were strongly integrated in the network, considering that the top miRNAs in terms of fold change (yellow border triangles) showed interactions with key molecules altered in kidney injury (blue and orange rectangles) (Figure S6A in Supplementary Material). Moreover, using a functional and enrichment approach, we verified in this miRNA–mRNA network that the top miRNAs in fold change (yellow border triangles) also interacted with specific genes involved in TGF-β/Wnt (purple border rectangles) and fibrosis/epithelial–mesenchymal transition (EMT) (orange rectangles) signaling pathways (Figure S6A in Supplementary Material). Furthermore, to heighten our inference analysis, we compared in a Venn diagram all the miRNA profiles and selected the 10 miRNAs (overlapping*) that were exclusively related to MSC cytoprotection (Figure S6B in Supplementary Material). Interestingly, we also identified in this overlap two miRNAs (i.e., miR-141 and miR-377) that were inversely regulated between the “*injury profile*” (Cis vs. Saline) vs. the “*treatment profile*” (Cis + MSC vs. Cis) (Figure 4D; Figure S6B in Supplementary Material). After, using these 10 pre-selected miRNAs, we constructed a second miRNA–mRNA network (“*regulatory network*”) and identified their corresponding predicted target genes (Figure 5A). Then, we selected only the genes that were targeted by at least two miRNAs in order to visualize the most relevant miRNA–mRNA interactions (Figure 5A). In this second regulatory network, the most connected miRNAs were over again the miR-141 and miR-377 (yellow border triangles) (Figure 5A). Subsequently, after enrichment analysis, we extracted semantic relationships between these miRNAs and again verified connection with genes related to the TGF-β/Wnt (purple border rectangles) and fibrosis/EMT (orange rectangles) signaling pathways (Figure 5A). Furthermore, in search of specific functions associated to these most connected miRNAs (e.g., miR-141 and miR-377), we built a second Venn diagram with additional putative target genes predicted by three distinct databases, i.e., Targetscan, miRanda, and miRDB (Figure 5B). Finally, the results from this second Venn diagram revealed three potential target genes for miR-141 (i.e., Yaf2, Ulk2, and Ccne2) and eight potential target molecules for miR-377 (i.e., Ncoa6, Bend6, Nts, Cul1, Pitx2, Zfp36l1, Ssfa2, and Rsbn1) (Figure 5B). Thus, based on its biologic importance and considering the renal context of injury and repair, only the most representative target genes (i.e., Ulk2 and Cul1) were selected to validation step analysis.

### miRNAs Actively Participate in Tissue Injury and Repair during AKI

**Figure 6:**
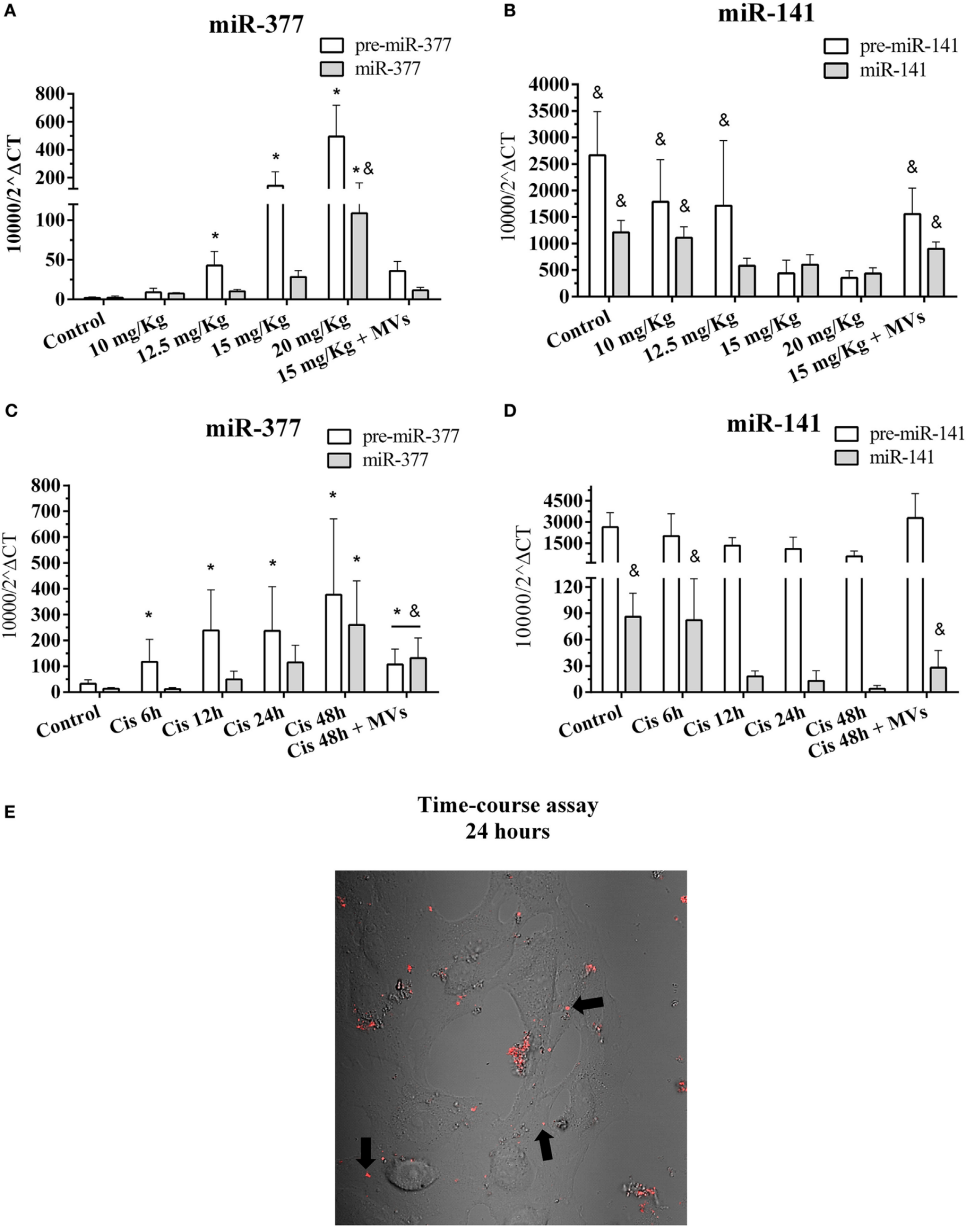
**Kinetics of expression of the top differentially expressed pre- and mature miRNAs after MSC or MV treatments and MVs incorporation**. **(A)** Expression of mature and pre-miR-377 in renal tissues with progressive damage by cisplatin; **(B)** expression of mature and pre-miRNA-141 in renal tissues with progressive damage by cisplatin; **(C)** expression of mature and pre-miR-377 in MM55.K tubular cells with progressive damage by cisplatin and MVs treatment; **(D)** expression of mature and pre-miR-141 in MM55.K tubular cells with progressive damage by cisplatin and MVs treatment; and **(E)** progressive time-course assay of *in vitro* MVs incorporation (dim red dots and black arrows) into tubular epithelial cells per 24 h, also demonstrated in Movie S3 in Supplementary Material (*p < 0.05 compared with the saline/control group and ^&^*p* < 0.05 compared with the cisplatin treatment, 15 and 20 mg/kg, 24 and 48 h). Cis, cisplatin; MSC, mesenchymal stromal cell; MVs, microvesicles.

To validate the participation of miR-377 and miR-141 in tissue injury and repair, we evaluated the dynamic expression of the precursor and mature forms of these miRNAs. Cisplatin treatment *in vivo* increased both mature and pre-miR-377 levels, proportionally to the damage severity (Figure 6A). Conversely, the renal tissue expression of both mature and pre-miR-141 species was higher when the injury was absent and lower when the damage process was already established (Figure 6B). Conforming observed at MSCs treatment in our global miRNA profile (Cis + MSCs vs. Cis), the treatment with MVs also reduced abruptly the miR-377 expression and increased the levels of miR-141 in renal tissues submitted to toxic injury (Figures 6A,B). Interestingly, the detection of the pre-miR-377 in early stages of the renal tissue injury suggests that its expression could be used as a potential marker of AKI (Figure 6A). Concordantly, we observed similar results using tubular cells *in vitro*, which showed elevated expression of mature miR-377 during toxic injury progression, considering that the levels of mature miR-141 decreased at the same time points (Figures 6C,D). Surprisingly, we also observed *in vitro* that treatment with MVs was able to decrease miR-377 expression and increase the miR-141 levels when compared to cisplatin-treated cells alone (Figures 6C,D). According to the damage severity, we also observed *in vitro* a premature expression of pre-miR-377 than its mature form (miR-377), whereas the pre-miR-141 expression remained unaltered (Figures 6C,D). For elucidating the incorporation process of MVs by tubular epithelial cells, we added pre-stained MVs suspensions (MVs labeled with fluorescent red dye) to injured tubular cells cultures (cultured with cisplatin) and analyzed its progressive incorporation per 24 h in a time-lapse assay. We observed that MVs, although in aggregated status, are internalized by epithelial tubular cells (dim red dots and black arrows) with predominant localization in the cytoplasm (Figure 6E; Movie S3 in Supplementary Material).

### The miRNA–mRNA Interaction Plays a Fundamental Role in MSC-Mediated Protection

**Figure 7:**
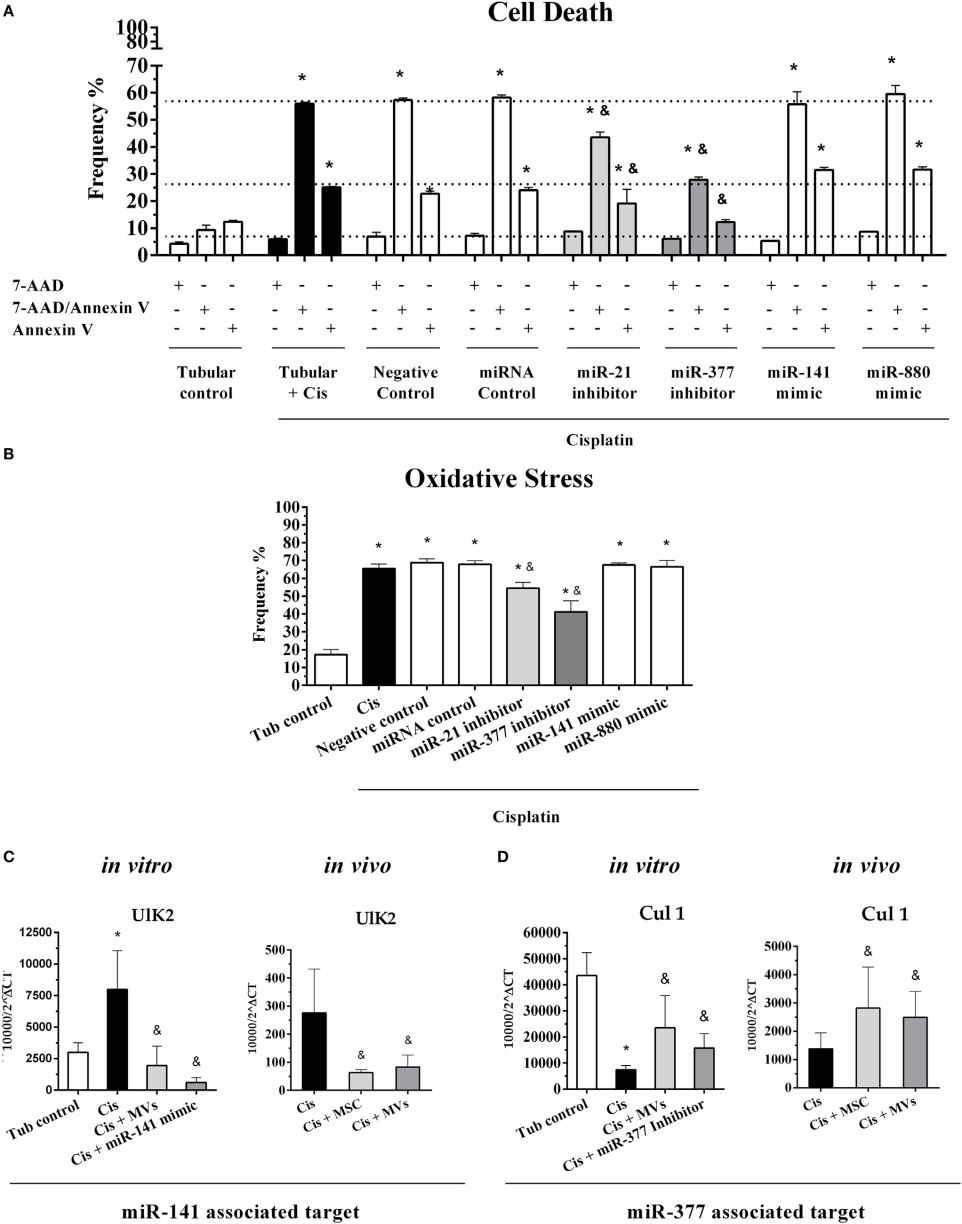
**Functional validation of differentially expressed miRNAs and their predicted targets**. **(A)** Evaluation of cell death frequency in MM55.K cells submitted to toxic injury with overexpression of miR-141 and miR-880 or silencing of miR-377 and miR-21 expressions; **(B)** evaluation of oxidative stress in MM55.K cells submitted to toxic injury with overexpression of miR-141 and miR-880 or silencing of miR-377 and miR-21 expressions; **(C)** validation *in vitro* and *in vivo* of the potential target of miR-141 (Ulk2), and **(D)** validation *in vitro* and *in vivo* of the potential target of miR-377 (Cul 1) (*p < 0.05 compared with the saline/control group and ^&^*p* < 0.05 compared with the cisplatin group). Cis, cisplatin; MSC, mesenchymal stromal cell; MVs, microvesicles; Tub, renal tubular cells.

To investigate the role of miRNA–mRNA interactions on kidney injury progression and tissue recover after MSC treatment, we conducted an *in vitro* transfection assay in tubular epithelial cells using specific oligos that work as mimics and inhibitors of some of the most relevant miRNAs found in our miRNA profile analysis (i.e., miR-880, miR-141 miR-377, and miR-21). First, we validated our transfection assays by demonstrating that all inhibitors and mimic oligos efficiently overexpressed or silenced their correspondent target molecules (Figure S7 in Supplementary Material). Then, we observed in tubular cells submitted to cisplatin treatment a protection against cell death (reduction in apoptosis/active caspase-3 and necrosis/7-AAD expression levels) only in cells incubated with inhibitors of miR-21 and miR-377 (Figure 7A). Complementary, the intracellular level of anion superoxide (${\text{O}}_{2}^{-},$ a stress oxidative radical) decreased only in cells treated with inhibitors of miR-21 and miR-377 (Figure 7B). By contrast, the use of miR-880 and miR-141 mimics, which were elevated in tissues treated with MSCs, did not show any direct protective effect on tubular cells submitted to toxic injury (Figures 7A,B). In addition, the *in vitro* inhibition of miR-377 in tubular epithelial cells promoted an increase in the expression of cytoprotective genes (i.e., BCL-2 and HO-1; Figures S8A,B in Supplementary Material) and a reduction in damage-related molecules (i.e., Kim-1 and iNOS; Figures S8C,D in Supplementary Material). The absence of any significant effects in the control (cells without cisplatin stimulus), the negative control (cells only exposed to transfection reagents), and the scrambled oligos groups (miRNA control, cells treated with not associated miRNAs) corroborated the relevance of our results (Figures 7A,B; Figures S7A–D and S8A–D in Supplementary Material). In sequence, we selected potential target genes for miR-141 (i.e., Ulk2) and miR-377 (i.e., Cul1) and confirmed *in vitro* and *in vivo* their inverse relationships. Specifically, we identified *in vitro* that Ulk2 expression increased under injury conditions but after MVs treatment or miR-141 overexpression had its levels decreased when compared to cisplatin-treated cells alone (Figure 7C). These results were further confirmed *in vivo* by demonstrating that the Ulk2 levels were elevated in tissues damaged by cisplatin and decreased after MSC or MV treatment (Figure 7C). In parallel, we also showed *in vitro* that Cul1 expression was higher in normal conditions and decreased after cell injury (Figure 7D). Conversely, the *in vitro* Cul1 levels were enhanced after MV therapy or when miR-377 was silenced in comparison with cisplatin group alone (Figure 7D). Finally, *in vivo* measurements showed that Cul1 levels were lower during AKI but increased in renal tissues after MSC or MV administration (Figure 7D).

## Discussion

In order to maintain tissue turnover, the cross-talk between cells is a required and organized process that contributes significantly to horizontal molecular exchange (25). MVs are emerging as key mediators of cell-to-cell communication that underpin the maintenance of physiological processes and tissue development (26). Thus, the concept that MVs can modulate the fate of target cells *via* the genetic transfer of information is an emerging paradigm in regenerative medicine (27).

In an attempt to investigate this mechanism, we hypothesized that MSCs mediate tissue repair by secreting MVs that transcriptionally modulate the molecular pattern in injured renal cells. Initially, using an experimental model of cisplatin-induced AKI, we demonstrated *in vivo* and *in vitro* that MVs, although by distinct mechanisms, mimic the beneficial effects of MSC treatment modulating a prosurvival program and inhibiting cell death. Furthermore, we identified a prominent participation of miRNAs as main protagonists in the regulation of this protective effect.

In this sense, the miRNA profile has been characterized in our study and we verified a distinct pattern between MSCs and MSC-MV profiles. Moreover, the enriched miRNAs inside MVs were not directly associated with most regulated miRNAs in renal tissues suggesting an indirect regulatory role of MSCs/MVs-derived miRNAs. A recent work have conducted a GO analysis of predicted and validated targets of highly expressed miRNAs in MSCs and MVs, and the authors demonstrated that these modulated miRNAs could be associated with the control of multi-system and -organ development, immune system, cellular differentiation, and cell survival (6). In fact, we showed here, *in vitro* and *in vivo*, that markers related to cytoprotection, oxidative stress, apoptosis, and inflammation were modulated after MSCs and MVs therapies. In this landscape, we analyzed the global miRNA signature in renal tissues after MSC treatment and observed at the predicted target genes of the most differentially expressed miRNAs a preferential association with ErbB, P13K-Akt, Wnt, and MAPK signaling pathways (Figure 4E).

These pathways play an essential role in tissue recover and development, as well as coordinate cellular health physiology. Indeed, a previous study from our group already demonstrated the participation of MAPK, ErbB, and Wnt signaling pathways during renal damage progression and their modulation after treatment with cytoprotective and anti-oxidant molecules (28). Moreover, different studies have established an interesting interplay between the Notch–PI3K/Akt and mTOR–PI3K/Akt pathways in the regulation of renal cells physiology (29–31). In line with this evidence, our results here demonstrated a strict association between differentially modulated miRNAs with regulatory pathways linked to positive modulation of transcription-dependent biological process, thereby highlighting the role of MSCs and MVs to restore normal renal function after AKI.

Furthermore, we were able to correlate the miRNA changes in cisplatin-induced AKI with published mRNA alterations that had been previously linked to cisplatin damage. Interestingly, this first miRNA–mRNA network, “*injured network*,” related to toxic injury was mainly associated with alterations in TGF-β/Wnt and fibrosis/EMT signaling pathways. Subsequently, our second mRNA–miRNA network, “*regulatory network*,” which was comprised by 10 pre-selected miRNAs found in the overlapping (Figure S6B in Supplementary Material), showed again a close link between these miRNAs with genes enriched in the TGF-β/Wnt and fibrosis/EMT signaling pathways. This evidence is in accordance with previous results describing the reciprocal interactions between miRNAs and Wnt genes during kidney disease progression (32). The Wnt pathway is involved in development, injury repair, tissue homeostasis, and progressive fibrosis (33, 34). Indeed, the activation of canonical Wnt signaling is required for TGF-β-mediated fibrosis (35). Fibrosis is related to a chronic pathologic status, and it is postulated that EMT is a potent pro-fibrotic process, which primarily is activated by TGF-β (36). Thus, considering a context of toxic AKI, the search for Wnt/TGF-β related targets may be an interesting strategy to understand how MSCs elicit their therapeutic properties.

Complementarily, the use of miRNA–mRNA network analysis allowed us to understand, in a systematic and integrative perspective, how miRNAs are linked to their specific target genes. Additionally, we identified miR-377 and miR-141 as the most connected miRNAs in the MSC-modulated network (*regulatory network*), thereby indicating their eminent participation in the MSC-protective effect against renal toxic damage. In agreement with this, we found *in vitro* and *in vivo* that both pre- and mature miRNAs corresponding species were inversely regulated during AKI and had their levels normalized after MSC or MV administration. Then, using specific silence oligos, we revealed *in vitro* the direct participation of miR-337 in the mediation of cell death and oxidative stress process in injured tubular cell. In particular, miR-377 is overexpressed in renal chronic disease and its expression is associated with fibronectin accumulation (37). In fact, TGF-β expression positively regulates miR-377 in diabetic nephropathy (38). Moreover, miR-377 promotes matrix deposition and thereby directly contributes to pro-fibrotic response elicited by TGF-β (39). Conversely, the miR-141 levels are reduced in renal disease and cancer (40, 41). Additionally, miR-141 expression is negatively regulated by TGF-β, which promotes EMT and fibrosis *via* a Smad-dependent signal (42). Taken together, these results suggest that the inverse regulation of miR-377 and miR-141 may represent the molecular mechanisms underlying to MSC protection, considering here a context of cisplatin-induced kidney injury.

To document this interesting connection, we identified putative targets genes for these specific miRNAs (i.e., miR-377 and miR-141) and evaluated their expression *in vitro* and *in vivo*. We showed that Ulk2 (predicted target of miR-141) is widely regulated by miR-141 and Cul1 (predicted target of miR-377) level is directly affected by miR-377 expression. Cul1 is fundamental to normal embryogenesis and participates in ubiquitin proteolytic system regulation, whereas its deletion in mice causes the accumulation of cyclin E, which leads to cell-cycle arrest and chromosome instability (43, 44). The essential function of Ulk2 is not entirely elucidated, but studies have suggested its evolutionarily participation in the regulation of autophagy, cytoskeleton reorganization, vesicle traffics, and adaptation to stress stimulus, including starvation (45–47).

The precise mechanism whereby MSC protects tissue injury can be designed as “*many-worlds interpretation*” process; however, here we demonstrated that the interplay between miRNAs and their mRNA targets represents a new and unexplored mechanism by which MSCs can restore kidney tissue function. In summary, our experimental validation and *in silico* prediction suggest that miRNAs conveyed by MSC-MVs could participate in the modulation of an inner cell program in target damage renal cells re-establishing tissue global homeostasis and system recovery. Thus, the fine tuning of miRNA–mRNA regulatory networks may underlie some of the protective effects induced by MSCs or MVs in the renal tissues providing a better understanding of kidney injury/recover process. Furthermore, we believe that our main findings illustrated here may contribute substantially for development of new and prospective therapeutic strategies for acute and chronic kidney diseases.

## Author Contributions

Conceptualization: DA and ÊB; investigation and resources: DA, HA, LA, CO, MC, VA-O, RF, RS, and MH; supervision: PS, CM-F, SV-A, ÁP-S, and NC; writing: DA and NC.

## Conflict of Interest Statement

The authors declare that the research was conducted in the absence of any commercial or financial relationships that could be construed as a potential conflict of interest. The reviewer TU and handling Editor declared their shared affiliation, and the handling Editor states that the process nevertheless met the standards of a fair and objective review.

## Supplementary Material

Movie S1 **Movement pattern visualization of animal treated with cisplatin (injured group)**. Movie file (.avi).

Movie S2 **Movement pattern visualization of animal treated with cisplatin and MSCs (treated group)**. Movie file (.avi).

Movie S3 ***In vitro* MVs incorporation into tubular epithelial cells**. Movie file (.avi).
