# Supplementary material for: A Regulatory miRNA–mRNA Network Is Associated with Tissue Repair Induced by Mesenchymal Stromal Cells in Acute Kidney Injury
Source: Front Immunol. 2017 Jan 3;7:645. doi: 10.3389/fimmu.2016.00645 (PMC5206861; doi:10.3389/fimmu.2016.00645)
Supplement: Supplementary file 4 [file Presentation_2.PDF]

Supplementary Material

A regulatory miRNA-mRNA network is associated with tissue repair induced by mesenchymal stromal cells in acute kidney injury

Danilo Candido de Almeida, Ênio Jose Bassi, Hatylas Azevedo, Letícia Anderson, Clarice Silvia Taemi Origassa, Marcos Antônio Cenedeze, Vinicius de Andrade-Oliveira, Raphael José Ferreira Felizardo, Reinaldo Correia da Silva, Meire Ioshie Hiyane, Patricia Semedo, Marlene Antônia dos Reis, Carlos Alberto Moreira-Filho, Sergio Verjovski-Almeida, Álvaro Pacheco-Silva and Niels Olsen Saraiva Câmara.

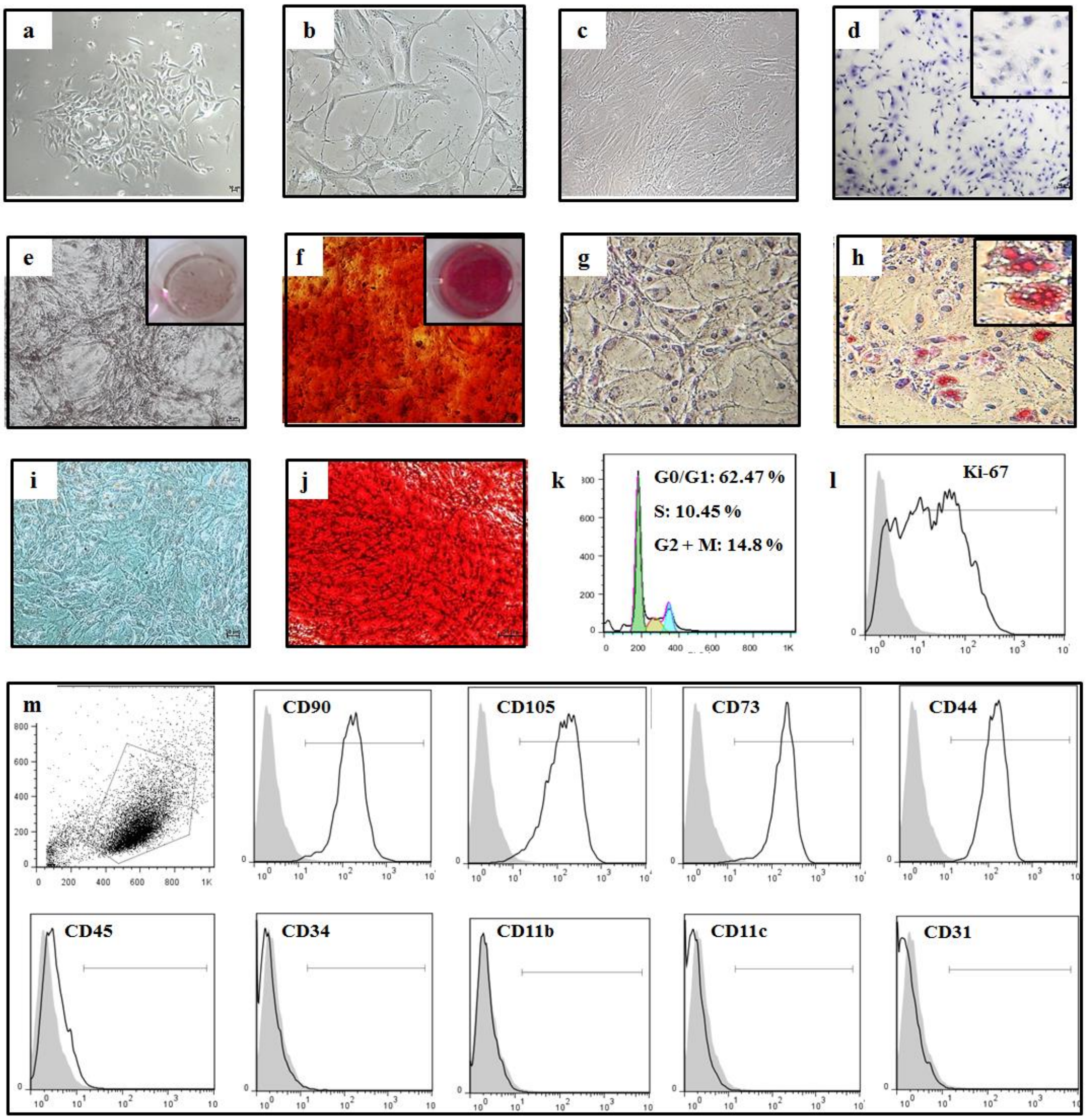

Figure S1. Characterization of adipose-derived MSCs, related to Figure 1.

(**a**) Fibroblastic colony formation (CFU-F); (**b**) Fibroblastic morphology; (**c**) High onfluence expansion; (**d**) MSCs stained with Harris hematoxylin; (**e-f**) Undifferentiated and differentiated MSCs for osteoblasts differentiation, respectively; (**g-h**) Undifferentiated and differentiated MSCs for adipocyte differentiation, respectively; (**i-j**) Undifferentiated and differentiated MSCs for chondrocytes differentiation, respectively; (**k**) MSCs present active phases of the cell cycle (G2+M>10%); (**l**) MSCs have Ki-67 expression, a proliferative marker; (**m**) phenotypic characterization of MSCs to CD90, CD105, CD73, CD44 (positive markers) and CD45, CD34, CD11b, CD11c and CD31 (negative markers); (**c-d**) Images with 40 X magnification, (**a-b**) and (**e-j**) Images with 200 X magnification.

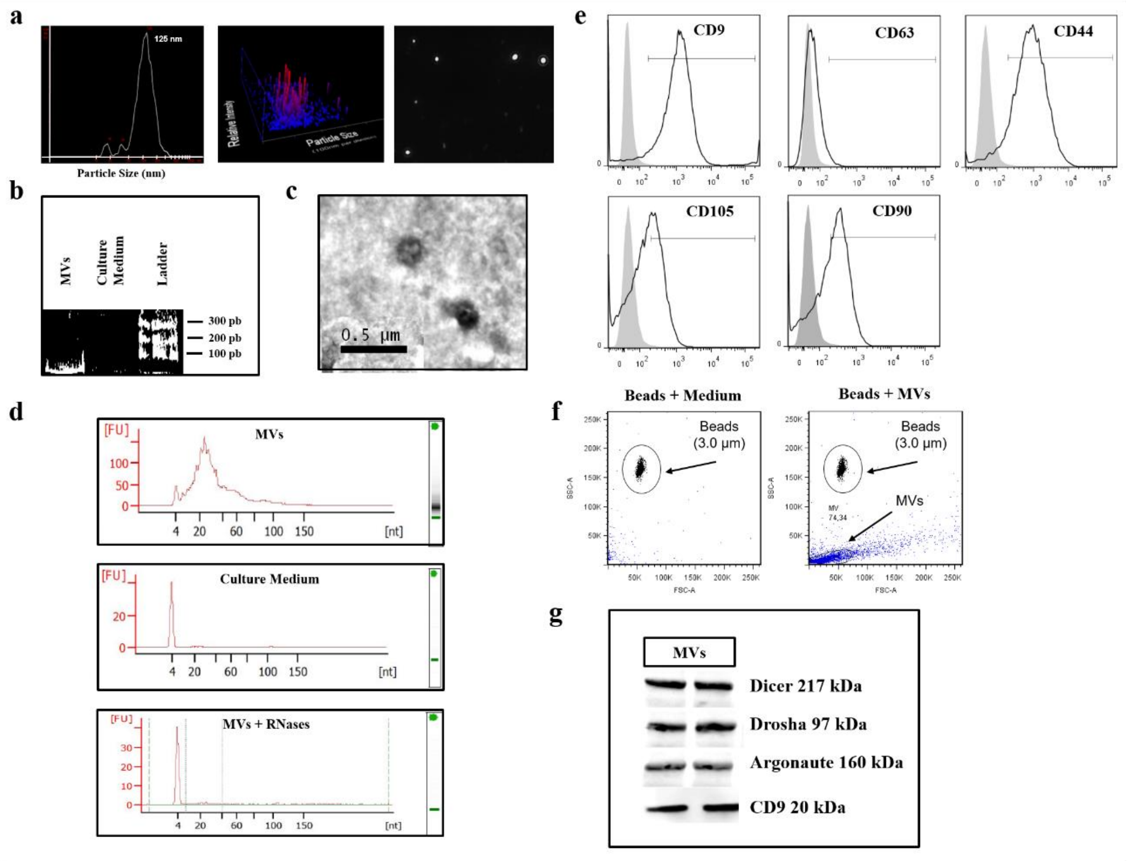

Figure S2. Characterization of MSC-derived MVs, related to Figure 1.

(**a**) Characterization of MVs by size using NanoSight instrument; (**b**) Evaluation of ribonucleic acid content from extracted fraction of MVs in 15% polyacrylamide gel; (**c**) Electron microscopy image showing the spheroid morphology of MVs; (**d**) Capillary electrophoresis gel of MVs suspension observing a predominant fraction of molecules with 20 nucleotides correspondent to small RNAs (miRNAs); (**e**) Phenotypic characterization of MVs tested by positive markers such as CD9 and CD63 and markers of host cells such as CD90, CD105, and CD44; (**f**) Flow cytometry comparison of MVs and beads with pre-defined size (3µm); and (**g**) Western Blotting of MVs fraction showing the presence of MVs biomarker CD9 and molecules related to miRNA biogenesis (e.g. Dicer, Drosha and Argonaute 2).

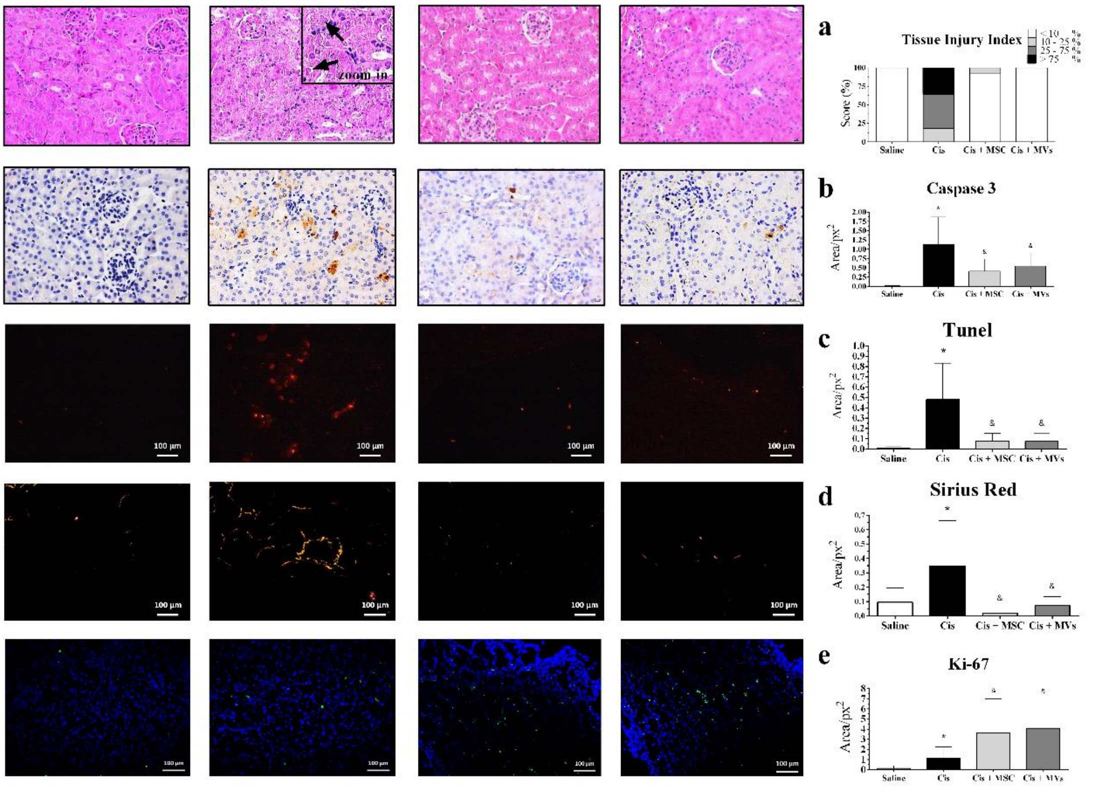

Figure S3. MSC and MV administration protected tissues from toxic damage, related to Figure 2.

(**a**) Histopathologic evaluation by hematoxylin and eosin staining (HE); (**b**) Immunohistochemistry for caspase-3, an apoptosis marker; (**c**) Fluorescence *in situ* assay for detection of necrosis by the TUNEL method; (**d**) Detection of fibrosis score by sirius red staining using polarized light microscope; (**e**) Immunofluorescence for Ki-67, a cell proliferation marker. (* $p<0.05$ compared with the saline/control group and $^{\&}$ $p<0.05$ compared with the cisplatin group). Cis: cisplatin, MSC: mesenchymal stromal cells and MVs: microvesicles.

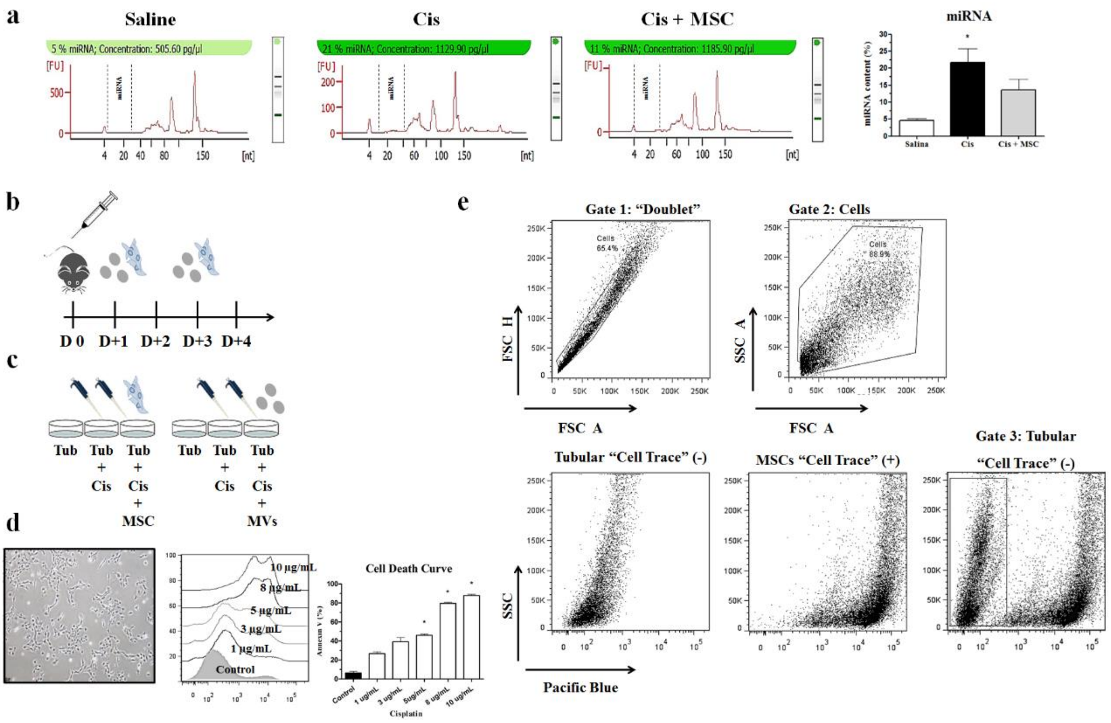

Figure S4. Strategies utilized to *in vitro* and *in vivo* assays using MSCs and MVs treatment, related to Figure 1, 2 and 3.

(**a**) Evaluation of miRNA content in renal tissues treated or not with cisplatin or MSC; (**b**) Schematic illustration of the infusion of MSC and MVs in animals previously injected with cisplatin; (**c**) Schematic illustration of *in vitro* assays using co-cultures with MSCs and MVs; (**d**) Dose response curve of cisplatin toxicity in tubular epithelial cells cultures and (**e**) Gates strategy used to separate MSC from tubular epithelial cells in co-culture experiments during flow cytometry analysis (rectangular gate refer to tubular cell, "cell trace negative").

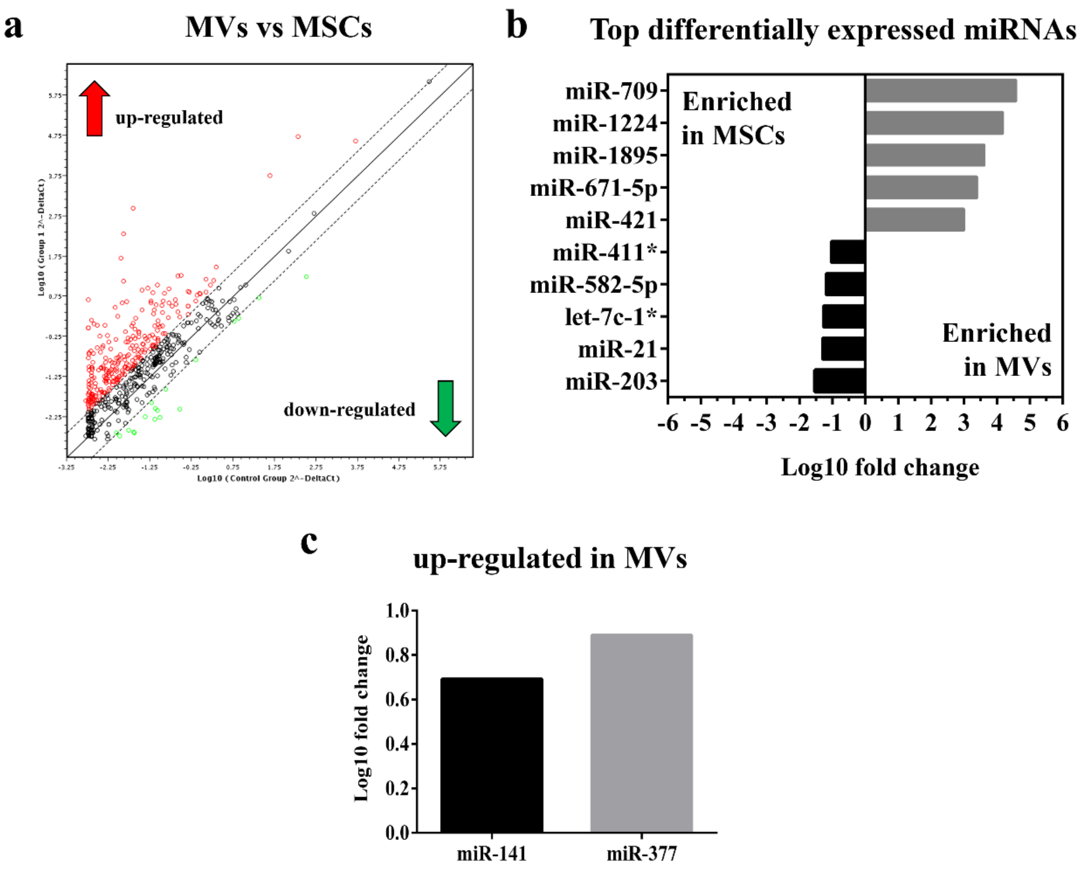

Figure S5. Global miRNA profile of MSCs and MSCs-derived microvesicles, related to Figure 4.

(**a**) Scatter plot analysis of differentially expressed miRNAs between MSCs and MVs; (**b**) Expression levels of top differentially expressed miRNAs in MSCs and in MVs; and (**c**) MVs expression level of most regulated miRNAs found in renal tissues (i.e. miR-141 and miR-377).

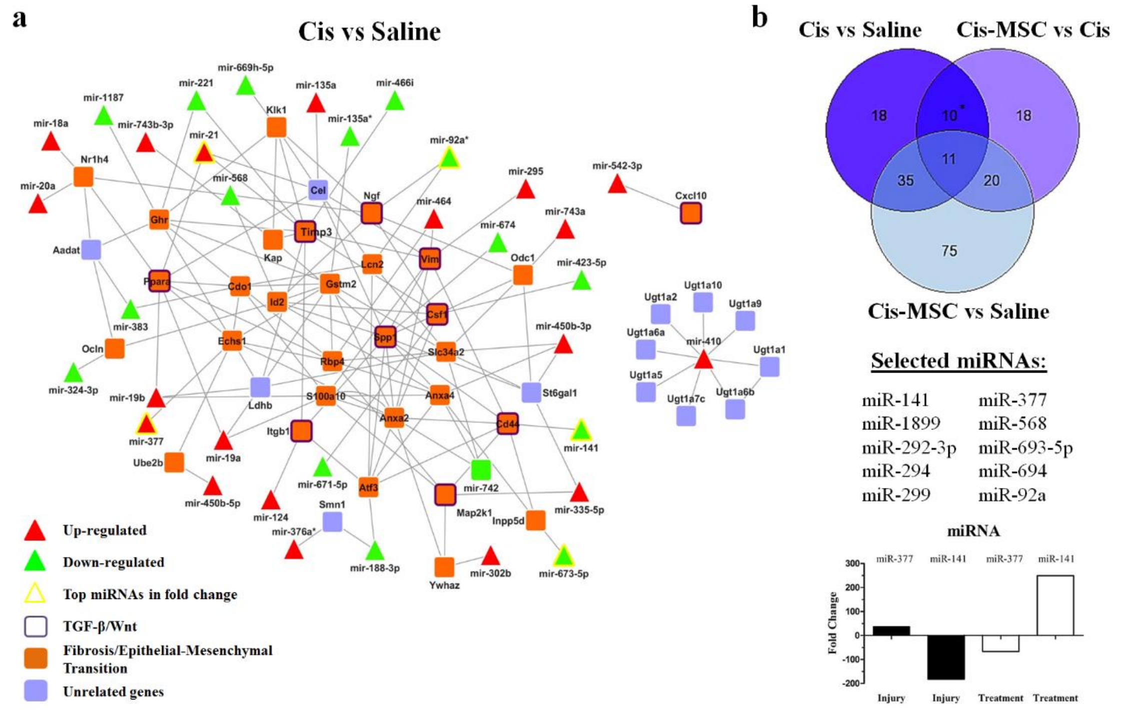

Figure S6. miRNA-mRNA network in cisplatin-induced toxic AKI and top selected overlapping miRNAs in all miRNA profiles, related to Figure 5.

(**a**) miRNA-mRNA network (cisplatin-modulated network) showing the interactions between miRNAs regulated after the cisplatin challenge and genes whose expression was previously associated in the literature with cisplatin nephrotoxicity; and (**b**) Venn diagram representing the overlapping of differentially expressed miRNAs among all experimental groups (Cis vs saline, Cis-MSC vs Cis and Cis-MSC vs saline) and a list of 10 top selected microRNAs used to construct the subnetwork related to tissue recovery mediated by MSC treatment. Up- and down-regulated miRNAs are represented, respectively as red and green triangles. The top five miRNAs in terms of fold changes had their border colors altered to yellow. Genes previously linked to fibrosis or EMT are represented as orange rectangles. Genes that were previously related to the TGF-β or Wnt pathways additionally had their border colors altered to purple and unrelated genes are represented by blue rectangles. Cis: cisplatin, MSC: mesenchymal stromal cells and MVs: microvesicles.

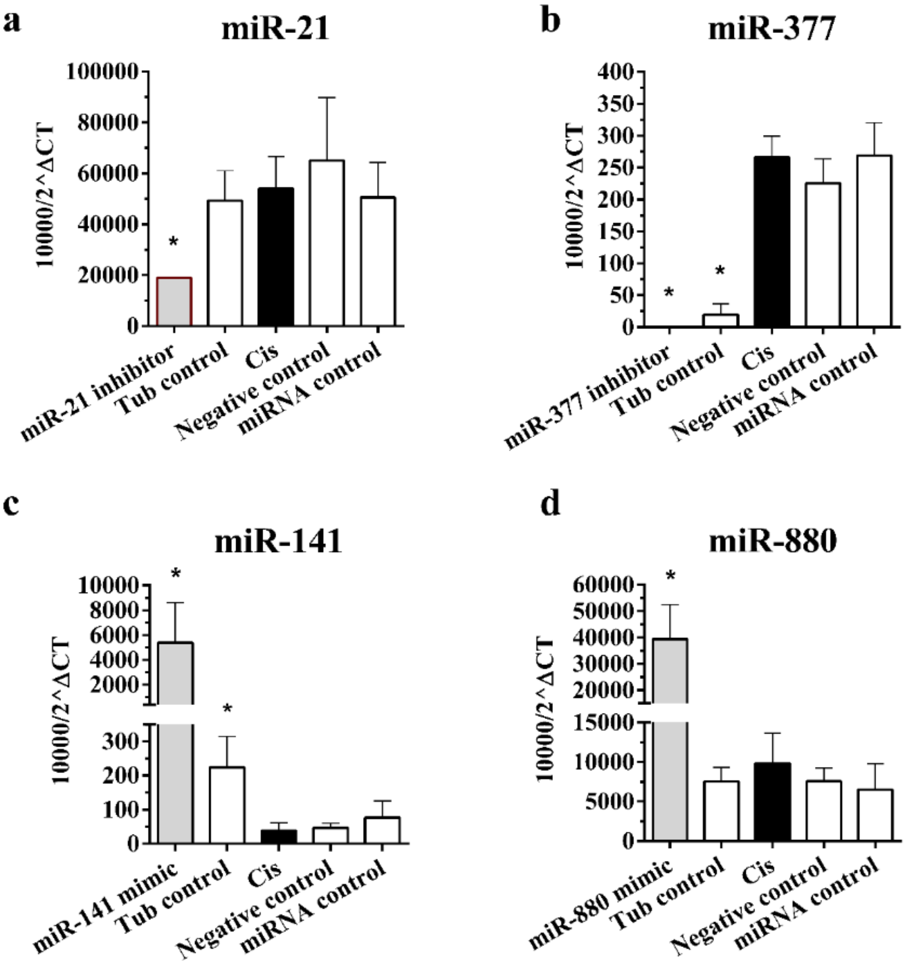

Figure S7. Validation assays of mimics and inhibitors oligos of selected miRNAs in transfected tubular cells cultures, related to Figure 7.

(**a**) miR-21 expression after miR-21 specific inhibitors treatment; (**b**) miR-377 expression after miR-377 specific inhibitors treatment; (**c**) miR-141 expression after miR-141 mimic treatment; and (**d**) miR-880 expression after miR-880 mimic treatment. (* *Tukey post-test*, $p<0.05$ compared to all groups).

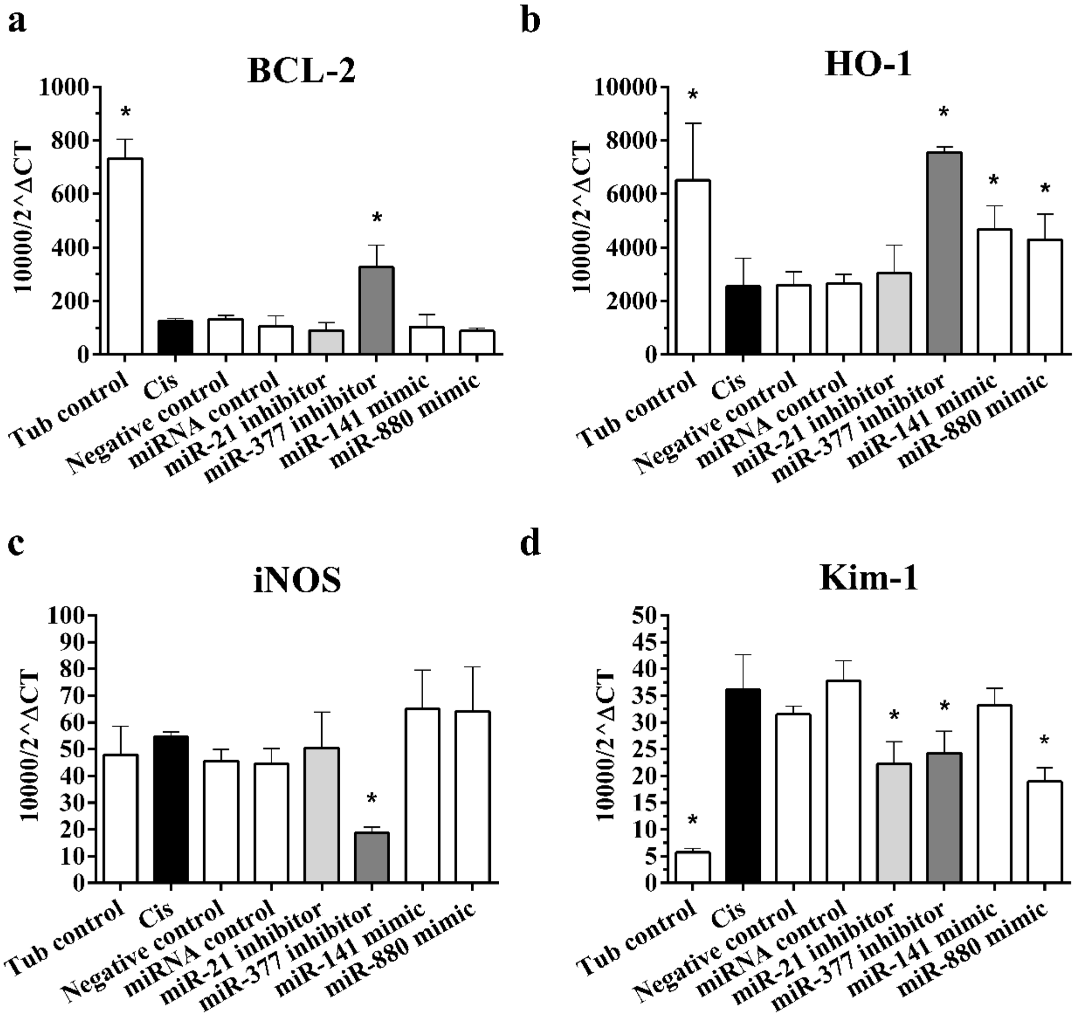

Figure S8. Gene expression profile of molecules associated with kidney injury and repair, related to Figure 7.

(**a**) BCL-2 expression in transfected cultures of tubular epithelial cells treated with mimics or inhibitors oligos of miRNAs; (**b**) HO-1 expression in transfected cultures of tubular epithelial cells treated with mimics or inhibitors oligos of miRNAs; (**c**) iNOS expression in transfected cultures of tubular epithelial cells treated with mimics or inhibitors oligos of miRNAs; and (**d**) Kim-1 expression in transfected cultures of tubular epithelial cells treated with mimics or inhibitors oligos of miRNAs. (* *Tukey post-test*, $p<0.05$ compared to cis group).

Table S1. Exemplification of all miRNAs provided in the miRNA array platform and its global fold changes according to each comparison.

		Global fold Change	
**miRNAs**	**Cis vs Saline**	**Cis + MSC vs Cis**	**Cis + MSC vs Saline**
**miR-142-5p**	17.172	29.756	51.097
**miR-16**	29.568	12.149	35.921
**miR-142-3p**	36.031	-12.167	29.613
**miR-21**	77.181	-13.893	55.556
**miR-124**	3.154	30.168	95.152
**miR-126-3p**	31.369	11.344	35.587
**miR-15a**	-13.444	-13.951	-18.756
**miR-29b**	-14.327	-11.839	-16.961
**miR-9**	22.484	-11.681	19.249
**let-7c**	-18.165	-19.635	-35.666
**miR-24**	15.046	-12.912	11.652
**miR-27a**	15.813	-12.794	1.236
**miR-30e**	13.608	-2268.921	-1667.349
**miR-22**	-11.792	-17.455	-20.583
**miR-30a**	21.398	11.698	25.032
**let-7a**	-16.973	-12.727	-21.602
**miR-30d**	15.999	1.334	21.343
**miR-140**	32.973	-11.543	28.565
**let-7f**	11.768	-12.393	-10.531
**miR-155**	17.435	-20.818	-1.194
**miR-130a**	1.096	-16.639	-15.182
**let-7b**	-18.042	-16.251	-2.932
**miR-322**	29.018	13.251	38.453
**miR-17**	25.058	-13.901	18.026
**miR-27b**	17.148	10.206	17.502
**miR-125b-5p**	20.429	1.536	31.379
**miR-29a**	-11.861	-21.332	-25.302
**miR-872**	25.164	1.189	2.992
**miR-32**	13.083	2.772	36.265
**miR-99b**	18.811	-12.376	0.00152
**miR-19b**	42.338	-14.412	29.376
**miR-191**	19.323	1.082	20.907
**miR-126-5p**	23.035	1.734	39.941
**miR-93**	18.244	-10.114	18.039
**miR-146a**	10.964	10.382	11.383
**miR-196b**	20.127	10.498	2.113
**let-7i**	-15.235	-18.371	-27.987
**miR-20a**	30.291	-14.885	20.351
**miR-18a**	30.967	-17.256	17.946
**miR-28**	18.585	-11.935	15.572
**miR-23b**	15.311	10.805	16.544
**miR-150**	14.751	10.297	15.189
**miR-92a**	-11.988	-11.598	-13.904
**miR-10a**	28.852	11.827	34.124
**let-7d**	-19.944	-13.979	-2.788
**miR-196a**	19.377	13.782	26.705
**miR-23a**	15.385	-10.557	14.573
**miR-106b**	17.763	-14.318	12.406
**miR-199b**	21.421	-15.063	14.221
**miR-34c**	11.759	15.641	18.393
**miR-503**	18.417	51.611	9.505
**miR-25**	18.106	1.138	20.605
**let-7g**	-12.954	-13.472	-17.452
**miR-96**	27.906	-15.551	17.945
**miR-31**	25.023	-16.668	15.013
**miR-30c**	16.139	11.742	1.895
**miR-15b**	-1.685	-10.593	-1.785

**miR-10b**	27.782	1.344	37.338
**miR-144**	11.713	14.758	17.286
**miR-467e**	19.834	10.068	19.969
**miR-125a-5p**	14.922	10.537	15.723
**miR-99a**	19.538	-12.614	15.489
**miR-880**	15.071	225.857	340.395
**miR-19a**	43.228	-14.184	30.475
**miR-199a-5p**	-12.916	-15.844	-20.465
**miR-488**	-11.418	13.856	12.136
**miR-182**	28.586	26.515	75.794
**miR-291a-3p**	-21.206	117.004	55.174
**miR-186**	12.571	-15.404	-12.253
**miR-541**	-10.392	61.309	58.995
**miR-302d**	10.901	120.942	131.842
**miR-183**	15.362	-11.334	13.555
**let-7e**	-15.226	-13.871	-21.119
**miR-140***	10.612	-10.246	10.357
**miR-411**	30.801	16.398	50.509
**miR-125b-3p**	-14.129	38.724	27.407
**miR-295**	63.676	13.964	88.917
**miR-1**	26.506	-12.201	21.724
**miR-214**	-10.002	10.075	10.073
**miR-138**	-14.607	15.597	10.678
**miR-425**	14.097	-14.163	-10.047
**miR-218**	14.876	10.215	15.196
**miR-335-5p**	38.512	11.956	46.045
**miR-101a**	21.903	12.142	26.594
**miR-141**	-182.428	2492.709	13.664
**miR-744**	17.837	-10.831	16.468
**miR-467c**	1.526	10.553	16.104
**miR-374**	28.399	15.723	44.652
**miR-134**	-12.051	1.165	-10.344
**miR-137**	22.513	-11.374	19.794
**miR-708**	10.249	-20.049	-19.562
**miR-181b**	-20.728	-15.392	-31.906
**miR-298**	-19.824	42.537	21.458
**miR-101b**	17.248	18.102	31.222
**miR-195**	20.584	22.297	45.897
**miR-29c**	12.163	-13.042	-10.722
**miR-300**	-12.442	11.724	-10.612
**miR-471**	16.283	12.833	20.895
**miR-122**	-17.848	2.159	12.097
**miR-302b**	46.541	96.133	447.406
**miR-30b**	15.889	18.823	29.909
**miR-33**	-42.583	20.207	-21.074
**miR-547**	13.376	23.613	31.584
**miR-130b**	-12.784	-12.289	-15.711
**miR-292-3p**	-151.444	69.739	-21.716
**miR-203**	19.382	23.969	46.458
**miR-301a**	20.443	15.872	32.448
**miR-431**	16.825	14.164	23.831
**miR-148a**	14.241	-10.889	13.079
**miR-375**	21.351	15.407	32.895
**miR-135b**	27.809	-10.845	25.641
**miR-449a**	-19.419	-1.307	-25.381
**miR-20b**	2.014	1.122	22.598
**miR-151-5p**	10.953	16.825	18.428
**miR-152**	15.738	15.205	23.929
**miR-103**	-17.343	10.599	-16.363
**miR-34a**	18.186	-21.193	-11.653
**miR-7a**	17.927	11.624	20.839
**miR-377**	362.859	-665.642	-18.344
**miR-652**	-22.146	-17.364	-38.454

**miR-9***	30.018	22.144	66.473
**miR-381**	-23.941	11.363	-2.107
**miR-193**	-24.105	-11.565	-27.877
**miR-200a**	14.888	14.731	21.932
**miR-125a-3p**	-15.291	17.853	11.676
**miR-376a**	-10.563	13.754	13.021
**miR-378**	10.894	15.743	17.151
**miR-467a***	10.684	24.776	2.647
**miR-106a**	23.408	10.397	24.336
**miR-184**	18.555	-31.447	-16.948
**miR-291a-5p**	25.297	-21.459	11.789
**miR-147**	27.974	-23.126	12.096
**miR-128**	-16.222	11.204	-14.479
**miR-324-5p**	-13.391	1.027	-13.039
**miR-500**	-26.145	10.028	-26.073
**miR-185**	-18.452	1.026	-17.984
**miR-210**	-25.377	-10.706	-27.168
**miR-223**	16.229	18.349	29.777
**miR-21***	13.615	-1.235	11.024
**miR-301b**	0.00212	13.944	29.561
**miR-202-3p**	-16.857	31.259	18.543
**miR-208a**	-861.112	103.477	-83.218
**miR-324-3p**	-34.751	-19.773	-68.713
**miR-335-3p**	2.589	37.319	96.619
**miR-98**	1.039	12.271	12.749
**miR-153**	12.935	-17.995	-13.912
**miR-342-3p**	12.334	16.653	2.054
**miR-423-3p**	-16.388	-11.817	-19.366
**miR-194**	17.874	22.922	4.097
**miR-222**	-58.444	-18.368	-107.353
**miR-370**	-1.336	23.104	17.293
**miR-465a-5p**	-19.306	10.728	-17.996
**miR-100**	1.774	16.612	2.947
**miR-451**	-13.302	-15.304	-20.357
**miR-465a-3p**	11.619	15.039	17.473
**miR-878-3p**	-3.048	24.691	-12.345
**miR-320**	-19.428	-10.521	-20.439
**miR-188-5p**	-21.565	1.011	-21.332
**miR-192**	16.248	2.264	36.785
**miR-221**	-36.186	-27.414	-0.00992
**miR-423-5p**	-65.112	-16.984	-110.585
**miR-429**	2.193	15.099	33.111
**miR-672**	12.328	10.827	13.347
**miR-149**	-16.898	17.215	10.188
**miR-669a**	-13.976	1.333	-10.484
**miR-135a**	30.357	12.954	39.325
**miR-202-5p**	18.929	26.339	49.858
**miR-380-5p**	23.657	-23.284	1.016
**miR-470**	-10.807	12.681	11.734
**miR-129-5p**	10.743	16.152	17.352
**miR-290-5p**	22.104	37.105	82.018
**miR-292-5p**	-16.728	11.044	-15.147
**miR-361**	10.565	17.533	18.524
**miR-484**	-13.974	-11.625	-16.245
**miR-326**	16.228	16.825	27.304
**miR-350**	19.311	17.154	33.125
**miR-132**	22.732	12.391	28.166
**miR-299**	-51.442	31.332	-16.418
**miR-434-5p**	-11.108	14.699	13.233
**miR-674**	-38.926	-21.198	-82.517
**miR-133a**	-10.681	-20.451	-21.843
**miR-151-3p**	-13.247	17.521	13.226
**miR-181c**	-21.145	-17.873	-37.793

**miR-181d**	-13.486	-13.857	-18.687
**miR-296-3p**	11.341	21.277	24.131
**miR-369-5p**	14.734	27.238	40.132
**miR-148b**	10.979	-11.303	-10.295
**miR-296-5p**	-26.971	-18.185	-49.046
**miR-34b-5p**	13.419	-24.197	-18.032
**miR-363**	12.775	11.272	0.00144
**miR-421**	2.295	20.835	47.816
**miR-669c**	-27.715	-14.525	-40.254
**miR-466c-5p**	-24.998	10.449	-23.923
**miR-871**	-16.325	30.302	18.562
**miR-291b-3p**	-14.775	-14.357	-21.213
**miR-34b-3p**	14.795	15.106	2.235
**miR-351**	-14.049	12.067	-11.643
**miR-532-5p**	19.348	-11.246	17.205
**miR-201**	16.965	-6.843	-40.335
**miR-205**	25.653	-27.652	-10.779
**miR-339-5p**	-18.267	10.955	-16.675
**miR-497**	-13.742	-13.384	-18.392
**miR-574-3p**	10.708	21.089	22.582
**miR-743a**	35.012	12.486	43.716
**miR-107**	-18.694	11.163	-16.746
**miR-154***	21.854	-28.505	-13.043
**miR-331-5p**	-11.187	17.557	15.694
**miR-409-3p**	-11.262	12.818	11.381
**miR-466b-5p**	-15.936	11.281	-14.126
**miR-466f-5p**	-18.955	15.625	-12.131
**miR-146b**	21.275	15.823	33.664
**miR-330**	18.617	14.356	26.727
**miR-345-5p**	-25.635	-10.576	-27.111
**miR-380-3p**	15.193	25.518	3.877
**miR-384-5p**	26.679	-22.068	12.089
**miR-409-5p**	10.855	-12.033	-11.085
**miR-671-3p**	-13.443	18.452	13.726
**miR-673-5p**	-119.949	-20.078	-240.839
**miR-742**	-74.537	24.272	-30.709
**miR-877**	-12.585	19.724	15.673
**miR-883a-3p**	-15.526	13.651	-11.374
**miR-466d-3p**	11.824	22.118	26.153
**miR-129-3p**	-13.228	-1.005	-13.294
**miR-139-5p**	11.752	16.216	19.057
**miR-216a**	-1.839	21.789	11.848
**miR-219**	13.983	12.738	17.811
**miR-328**	-19.336	1.099	-17.594
**miR-342-5p**	1.525	-11.524	13.234
**miR-376b***	2.269	-16.945	1.339
**miR-384-3p**	12.801	-10.116	12.654
**miR-434-3p**	24.066	15.968	38.429
**miR-466a-5p**	-15.836	-33.175	-52.537
**miR-495**	15.342	-1.982	-12.919
**miR-542-5p**	1.653	11.957	19.764
**miR-542-3p**	32.349	22.583	73.053
**miR-802**	38.492	0.00212	81.602
**miR-188-3p**	-35.622	16.567	-21.502
**miR-19a***	24.204	-21.228	11.402
**miR-208b**	-5.561	-15.854	-88.165
**miR-290-3p**	-12.186	30.515	2.504
**miR-302a***	21.989	11.136	24.487
**miR-345-3p**	-12.162	13.124	10.791
**miR-362-3p**	13.639	10.759	14.674
**miR-466d-5p**	-23.325	10.926	-21.348
**miR-466f-3p**	-25.181	11.734	-21.461
**miR-501-3p**	-10.243	14.807	14.455

**miR-615-3p**	-18.811	-13.068	-24.581
**miR-665**	-1.402	24.889	17.752
**miR-666-3p**	18.072	-16.289	11.095
**miR-92b**	13.946	19.719	27.499
**miR-187**	-13.902	11.602	-11.982
**miR-217**	-16.409	22.306	13.594
**miR-224**	12.534	-11.065	11.328
**miR-294***	-93.586	61.933	-15.111
**miR-302c***	10.483	25.603	26.838
**miR-331-3p**	-19.708	-10.469	-20.632
**miR-337-5p**	0.00116	11.748	13.628
**miR-433**	2.087	-37.284	-17.865
**miR-450a-5p**	26.021	17.604	45.808
**miR-455**	-15.803	10.813	-14.614
**miR-485**	-17.086	11.797	-14.483
**miR-487b**	-54.358	-21.168	-115.067
**miR-543**	1.225	-121.991	-99.583
**miR-883a-5p**	-18.068	12.458	-14.503
**miR-133b**	-1.924	12.862	-14.959
**miR-212**	18.095	17.752	32.122
**miR-325**	28.429	10.048	28.566
**miR-337-3p**	14.253	14.787	21.076
**miR-340-3p**	16.104	2.532	40.776
**miR-341**	-4.043	-12.137	-49.071
**miR-362-5p**	19.045	0.00152	28.948
**miR-382**	12.799	14.158	18.121
**miR-383**	-63.598	10.569	-60.175
**miR-450a-3p**	-19.514	34.045	17.447
**miR-450b-3p**	30.125	28.987	87.324
**miR-466g**	-37.261	14.239	-26.169
**miR-532-3p**	-13.413	1.453	10.833
**miR-574-5p**	-72.226	-11.473	-82.869
**miR-743b-5p**	22.758	1.383	31.473
**miR-760**	-23.395	21.408	-10.928
**miR-770-5p**	24.024	2.703	64.936
**miR-770-3p**	11.854	29.949	35.503
**miR-874**	-2.772	1.513	-18.321
**miR-127***	-15.007	12.937	-0.00116
**miR-139-3p**	29.375	24.249	71.232
**miR-190b**	2.734	1.576	43.088
**miR-193b**	-11.723	11.765	10.035
**miR-197**	13.855	15.494	21.466
**miR-200b***	-10.975	16.845	15.349
**miR-200c***	13.551	15.564	21.091
**miR-216b**	15.883	13.465	21.387
**miR-26b**	16.279	19.061	31.029
**miR-323-5p**	11.148	14.192	15.821
**miR-329**	-35.668	13.461	-26.498
**miR-338-5p**	-12.122	22.352	18.439
**miR-338-3p**	18.163	11.384	20.676
**miR-483***	-1.435	-18.241	-26.177
**miR-486**	-1.627	11.799	-13.789
**miR-489**	18.565	16.815	31.217
**miR-501-5p**	-13.802	11.406	-12.101
**miR-582-5p**	17.602	13.733	24.172
**miR-582-3p**	1.892	21.941	41.512
**miR-590-3p**	11.409	52.202	59.557
**miR-7b**	17.006	10.098	17.172
**miR-876-3p**	12.168	19.263	23.439
**miR-882**	14.075	27.377	38.534
**miR-883b-5p**	1.513	19.009	2.876
**miR-883b-3p**	12.995	12.849	16.697
**miR-297b-3p**	-10.639	19.979	18.778

**miR-211**	-10.068	21.204	21.061
**miR-215**	24.683	27.919	68.913
**miR-220**	-11.327	13.292	11.735
**miR-327**	-19.672	50.709	25.778
**miR-343**	-0.0074	-14.032	-103.835
**miR-346**	-16.638	12.666	-13.136
**miR-448**	-14.279	16.458	11.526
**miR-449b**	12.697	18.776	2.384
**miR-452**	21.795	17.003	37.059
**miR-453**	-13.741	20.964	15.256
**miR-490**	-11.443	22.354	19.535
**miR-504**	-13.759	22.502	16.354
**miR-505**	12.686	15.518	19.687
**miR-509-5p**	-11.187	22.769	20.354
**miR-590-5p**	-12.632	0.00214	16.941
**miR-653**	28.186	26.392	74.388
**miR-654-3p**	18.921	31.652	59.888
**miR-654-5p**	-10.046	24.233	24.121
**miR-758**	18.244	28.322	5.167
**miR-875-3p**	-24.913	19.677	-12.661
**miR-875-5p**	18.415	34.671	63.847
**miR-876-5p**	25.341	30.774	77.984
**miR-881***	-15.627	10.997	-1.421
**miR-376c**	26.473	18.604	49.252
**miR-449c**	-13.042	16.706	12.809
**miR-464**	33.178	-20.748	15.991
**miR-467b***	10.451	17.577	1.837
**miR-496**	18.173	13.091	2.379
**miR-546**	-11.818	15.367	13.003
**miR-667**	12.988	29.316	38.076
**miR-669b**	-22.865	11.392	-20.071
**miR-675-5p**	12.361	-10.107	1.223
**miR-676**	-1.812	10.067	-17.999
**miR-677**	22.364	30.524	68.264
**miR-678**	19.277	17.308	33.365
**miR-679**	-16.718	-13.563	-22.675
**miR-681**	12.516	24.583	30.769
**miR-683**	17.903	11.787	21.102
**miR-684**	17.299	3.193	55.237
**miR-685**	22.663	16.611	37.645
**miR-686**	-26.479	23.179	-11.424
**miR-687**	-16.277	15.002	-1.085
**miR-688**	43.777	14.733	64.497
**let-7b***	-21.376	19.712	-10.844
**let-7c-1***	15.377	24.143	37.124
**let-7d***	14.998	0.00144	21.597
**let-7f***	12.257	21.366	26.188
**let-7i***	-22.178	-11.073	-24.557
**miR-106b***	-18.904	-11.808	-22.322
**miR-10b***	21.804	16.453	35.873
**miR-124***	48.896	17.255	84.369
**let-7g***	15.133	14.696	22.239
**miR-101a***	-11.695	14.618	12.499
**miR-1-2-as**	24.805	24.995	0.0062
**miR-130b***	11.117	12.693	14.111
**miR-133a***	17.221	-29.238	-16.978
**miR-135a***	-32.772	15.349	-21.351
**miR-138***	14.185	12.085	17.142
**miR-141***	24.109	-12.763	1.889
**miR-146b***	14.914	-17.647	-11.833
**miR-148a***	15.342	-42.008	-2.738
**miR-150***	-24.777	36.284	14.644
**miR-15a***	-29.201	-11.434	-33.388

**miR-15b***	23.042	18.191	41.916
**miR-16***	13.713	10.316	14.146
**miR-17***	-11.848	-14.636	-1.734
**miR-181a-1***	16.334	16.398	26.785
**miR-181a-2***	14.661	10.689	15.671
**miR-183***	17.717	-14.114	12.553
**miR-186***	10.985	-13.891	-12.646
**miR-18a***	10.342	-13.653	-13.202
**miR-191***	17.636	14.007	24.703
**miR-193***	-12.054	15.494	12.854
**miR-199b***	-1.641	-13.433	-22.044
**miR-203***	1.343	11.891	15.969
**miR-20a***	19.667	-11.565	17.006
**miR-20b***	13.419	19.911	26.717
**miR-218-1***	23.985	15.038	36.068
**miR-218-2***	31.248	25.228	78.834
**miR-22***	-15.847	-13.987	-22.165
**miR-24-1***	32.215	33.865	109.095
**miR-24-2***	1.684	-1.277	13.187
**miR-26b***	-11.934	-1.442	-17.209
**miR-28***	13.214	15.916	21.031
**miR-295***	-18.719	26.317	14.059
**miR-299***	20.852	-13.048	1.598
**miR-29a***	18.934	12.196	23.092
**miR-29b***	-10.725	-16.402	-17.592
**miR-29c***	-16.555	-10.914	-18.068
**miR-300***	-23.335	-20.401	-47.606
**miR-302b***	-14.867	10.208	-14.565
**miR-30a***	13.588	16.203	22.018
**miR-30b***	-1.425	-16.841	-23.999
**miR-30c-1***	-49.124	10.315	-47.624
**miR-30c-2***	-24.892	-15.523	-3.864
**miR-30e***	10.206	19.266	19.664
**miR-31***	41.997	-27.776	1.512
**miR-325***	-19.816	-1.112	-22.036
**miR-33***	10.977	15.839	17.386
**miR-330***	12.801	16.745	21.435
**miR-374***	-22.618	1.395	-16.214
**miR-376c***	-15.934	15.521	-10.266
**miR-378***	-24.149	-18.156	-43.846
**miR-382***	-29.676	34.125	11.499
**miR-411***	16.798	11.909	20.004
**miR-425***	-21.853	-14.115	-30.846
**miR-433***	-34.029	188.467	55.384
**miR-455***	2.721	17.779	48.375
**miR-466b-3-3p**	13.072	23.253	30.397
**miR-466i**	-31.405	15.918	-19.729
**miR-466j**	-14.464	20.635	14.267
**miR-466k**	-10.606	-10.904	-11.565
**miR-467b**	22.853	16.217	37.061
**miR-467f**	-1.296	19.523	15.065
**miR-467h**	-10.056	14.506	14.424
**miR-470***	-56.579	21.601	-26.193
**miR-485***	-15.132	10.865	-13.927
**miR-488***	-6.208	12.931	-48.007
**miR-503***	12.674	13.967	17.701
**miR-669d**	1.292	1.072	1.385
**miR-669e**	17.915	16.471	29.507
**miR-669f**	-11.379	17.111	15.038
**miR-669g**	10.372	16.414	17.024
**miR-669h-3p**	-11.746	12.635	10.757
**miR-669h-5p**	-42.885	13.154	-32.603
**miR-669i**	-18.171	-16.636	-3.023

**miR-669j**	10.633	1.149	12.218
**miR-669k**	-20.573	20.328	-1.012
**miR-7a***	27.149	14.489	39.336
**miR-92a***	-215.755	180.802	-11.933
**miR-99b***	11.002	13.152	1.447
**miR-105**	26.594	-30.611	-11.511
**miR-1186**	-49.067	-14.288	-7.011
**miR-1187**	-53.953	-10.732	-57.902
**miR-1188**	2.058	17.812	36.657
**miR-1190**	-107.489	-12.166	-130.769
**miR-1191**	16.685	15.323	25.567
**miR-1192**	-2.283	31.281	13.702
**miR-1193**	22.254	23.221	51.674
**miR-1194**	13.299	12.623	16.788
**miR-1195**	12.364	11.333	14.011
**miR-1196**	20.407	10.213	20.842
**miR-1198**	-15.768	12.525	-12.589
**miR-1199**	15.319	1.827	27.988
**miR-1224**	14.269	-12.755	11.187
**miR-145***	26.319	-11.317	23.256
**miR-145**	16.263	1.068	17.369
**miR-181a**	-18.887	-20.633	-38.969
**miR-1839-3p**	-22.098	14.527	-15.212
**miR-1892**	-14.112	33.684	23.869
**miR-1895**	-14.634	14.406	-10.158
**miR-1896**	-14.902	-10.531	-15.692
**miR-1897-3p**	-10.514	-1.255	-13.195
**miR-1897-5p**	21.917	-14.368	15.253
**miR-1898**	-17.568	46.424	26.425
**miR-1899**	-3.377	57.811	17.119
**miR-18b**	46.043	-11.717	39.296
**miR-1900**	-30.856	24.416	-12.638
**miR-190**	1.649	14.282	23.551
**miR-1928**	-13.712	-10.993	-15.073
**miR-196a***	22.422	11.259	25.246
**miR-200a***	17.413	20.269	35.296
**miR-200c**	22.468	13.368	30.035
**miR-204**	14.649	18.227	26.701
**miR-207**	15.479	13.406	20.751
**miR-26a**	14.376	16.043	23.063
**miR-27a***	23.139	13.057	30.213
**miR-293**	-15.194	-13.397	-20.355
**miR-293***	64.395	30.474	196.239
**miR-294**	-11.877	-10.854	-12.891
**miR-297b-5p**	-20.671	-20.338	-42.041
**miR-339-3p**	-12.831	1.373	10.701
**miR-340-5p**	21.545	14.511	31.263
**miR-365**	1.025	-10.894	-10.628
**miR-369-3p**	18.157	-11.521	1.576
**miR-376a***	41.882	14.631	61.279
**miR-376b**	25.816	-13.954	18.501
**miR-410**	31.164	-16.985	18.348
**miR-412**	2.089	14.124	29.505
**miR-450b-5p**	46.198	17.683	81.694
**miR-463***	20.139	129.179	260.153
**miR-463**	71.711	147.651	1058.825
**miR-469**	23.595	10.279	24.253
**miR-493**	-12.659	39.303	31.047
**miR-509-3p**	35.641	18.837	67.137
**miR-540-3p**	-12.428	43.042	34.633
**miR-544**	10.376	-10.098	10.276
**miR-551b**	26.102	10.349	27.014
**miR-568**	-44.954	97.237	2.163

**miR-598**	-11.524	-1.711	-19.717
**miR-615-5p**	-29.028	16.492	-17.602
**miR-666-5p**	12.117	18.771	22.745
**miR-669m**	10.531	43.393	45.698
**miR-671-5p**	-58.747	-36.485	-214.338
**miR-674***	13.031	15.471	20.161
**miR-675-3p**	8.575	2.184	187.275
**miR-676***	2.389	16.105	38.474
**miR-680**	28.856	24.437	70.515
**miR-682**	-14.071	40.466	28.757
**miR-1938**	3.624	47.315	17.147
**miR-692**	-12.306	-20.318	-25.003
**miR-693-3p**	13.697	-10.727	12.768
**miR-693-5p**	52.314	-88.616	-16.939
**miR-694**	-42.659	34.022	-12.539
**miR-695**	14.669	2.477	36.336
**miR-697**	-22.645	-13.526	-3.063
**miR-708***	10.553	-29.496	-27.952
**miR-709**	14.485	-12.437	11.647
**miR-712***	17.986	11.509	20.699
**miR-741**	89.314	-25.854	34.546
**miR-742***	-19.083	-12.707	-24.248
**miR-743b-3p**	35.697	3.055	109.057
**miR-744***	1.514	18.231	27.601
**miR-872***	17.866	17.563	31.377
**miR-877***	10.156	23.717	24.086
**miR-878-5p**	-14.798	43.968	29.713
**miR-879***	30.144	11.557	34.839
**miR-879**	-18.821	13.247	-14.208
**miR-93***	13.849	16.131	2.234

Table S2. Description of genes associated to signaling pathways linked to down and up regulated miRNAs in all comparisons.

Signaling Pathways					
**Up Regulated in Cis vs Saline Comparison**	Overall miRNAs Involved	Number of miRNAs	Overall target genes	Number of Targets	p valor
**Notch**	miR-693-5p, miR-21-5p and miR-295-5p	3	Aph1b, Aph1c, Dll1, Hes1, Jag1, Rbpj	6	0.0014
**PI3K-Akt**	miR-377-5p, miR-693-5p, miR-21-5p, miR-295-5p, miR-463-5p and miR-675-3p	6	Bcl2, Col1a1, Col5a1, Eif4e, Fasl, Fgfr2, Flt1, Gng5, Il2ra, Insr, Kit, Kras, Myb, Pik3r1, Pkn2, Ppp2r2a, Ppp2r5d, Ywhae	18	0.0027
**HIF-1**	miR-693-5p, miR-21-5p, miR-295-5p and miR-463-5p	4	Bcl2, Camk2a, Egln1, Eif4e, Flt1, Hk3, Insr, Pik3r1	8	0.0109
**Wnt**	miR-693-5p, miR-21-5p, miR-295-5p and miR-463-5p	4	Apc, Camk2a, Ctnnbip1, Dkk2, Mapk8, Nfatc3, Ppp2r5d, Ppp3cb, Prickle2, Rock1	10	0.0124
**Down Regulated in Cis vs Saline Comparison**	Overall miRNAs Involved	Number of miRNAs	Overall target genes	Number of Targets	p valor
**Wnt**	miR-141-5p, miR-208a-5p, miR-208b-5p, miR-222-5p, miR-292-3p, miR-299-5p, miR-423-5p, miR-673-5p, miR-742-5p, miR-1187, miR-1190, miR-383 and miR-671-5p	13	Apc, Btrc, Camk2a, Camk2b, Cer1, Csnk1a1, Csnk2a2, Ctbp2, Ctnnb1, Daam1, Dkk1, Dkk2, Fbxw11, Fzd4, Fzd9, Gsk3b, Lrp5, Lrp6, Map3k7, Mapk10, Mapk8, Nfat5, Nlk, Plcb1, Plcb2, Plcb4, Ppp2ca, Ppp2r5a, Ppp2r5c, Ppp2r5e, Ppp3cb, Prkca, Prkcg, Rhoa, Rock2, Sfrp1, Siah1a, Smad4, Tbl1x, Tbl1xr1, Tcf7l2, Vangl1, Vangl2, Wnt1, Wnt4, Wnt5a	46	1.06E-14
**ErbB**	miR-141-5p, miR-208a-5p, miR-208b-5p, miR-222-5p, miR-292-3p, miR-299-5p, miR-423-5p, miR-673-5p, miR-742-5p, miR-1187, miR-1190, miR-383 and miR-671-5p	13	Braf, Camk2a, Camk2b, Cdkn1a, Crk, Egfr, Erbb4, Gab1, Grb2, Gsk3b, Map2k1, Map2k7, Mapk10, Mapk8, Nras, Nrg3, Nrg4, Pak3, Pak7, Pik3ca, Pik3cg, Pik3r5, Plcg1, Prkca, Prkcg, Rps6kb1, Sos1, Sos2	28	9.76E-13
**PI3K-Akt**	miR-141-5p, miR-208a-5p, miR-208b-5p, miR-222-5p, miR-292-3p, miR-299-5p, miR-423-5p, miR-673-5p, miR-742-5p, miR-1187, miR-1190, miR-383 and miR-671-5p	13	Angpt4, Bcl2, Bcl2l11, Cdkn1a, Col11a1, Col4a4, Col4a6, Creb1, Csf1, Egfr, Fasl, Fgf1, Fgf10, Fgf14, Fgf16, Fgf23, Fgfr1, Fgfr2, Flt1, Flt4, Ghr, Gnb4, Gng12, Grb2, Gsk3b, Hsp90aa1, Igf1, Ikbkb, Il4ra, Itga11, Itga3, Itga4, Itga6, Itga8, Itga9, Itgav, Itgb4, Itgb8, Kdr, Lamc1, Lpar4, Map2k1, Mdm2, Nfkb1, Ngfr, Nras, Pck2, Pdgfb, Pdgfd, Pdpk1, Pik3ca, Pik3cg, Pik3r5, Ppp2ca, Ppp2r2a, Ppp2r2c, Ppp2r5a, Ppp2r5c, Ppp2r5e, Prkca, Pten, Rbl2, Rps6kb1, Rptor, Sgk1, Sos1, Sos2, Thbs3, Tsc1, Vegfa, Ywhag	71	3.80E-10
**MAPK**	miR-141-5p, miR-208a-5p, miR-208b-5p, miR-222-5p, miR-292-3p, miR-299-5p, miR-423-5p, miR-673-5pmiR-742-5p, miR-1187, miR-1190, miR-383 and miR-671-5p	13	B230120H23Rik, Braf, Cacna1h, Cacna2d2, Cacnb4, Cacng1, Crk, Daxx, Dusp2, Dusp7, Egfr, Fasl, Fgf1, Fgf10, Fgf14, Fgf16, Fgf23, Fgfr1, Fgfr2, Flnb, Fos, Gna12, Gng12, Grb2, Ikbkb, Map2k1, Map2k3, Map2k6, Map2k7, Map3k12, Map3k7, Map3k8, Map4k3, Mapk10, Mapk11, Mapk8, Mapt, Max, Mecom, Mef2c, Mras, Nfkb1, Nlk, Nras, Pdgfb, Ppp3cb, Prkca, Prkcg, Rapgef2, Rasa2, Rps6ka3, Rps6ka5, Sos1, Sos2, Taok1, Tgfb2, Tgfbr2	57	5.97E-09
**HIF-1**	miR-141-5p, miR-208a-5p, miR-208b-5p, miR-222-5p, miR-292-3pmiR-299-5p, miR-423-5p, miR-673-5p, miR-1187, miR-1190,	12	Angpt4, Arnt, Bcl2, Camk2a, Camk2b, Cdkn1a, Cul2, Egfr, Egln1, Flt1, Hk3, Igf1, Map2k1, Nfkb1, Pdha1, Pdk1, Pfkfb2, Pik3ca, Pik3cg, Pik3r5, Plcg1, Prkca, Prkcg, Rps6kb1, Slc2a1, Stat3,	27	2.16E-06

	miR-383 and miR-671-5p		Vegfa		
**mTOR**	miR-141-5p, miR-208a-5p, miR-208b-5p, miR-222-5p, miR-292-3p, miR-673-5p, miR-1187, miR-1190, miR-383 and miR-671-5p	10	Braf, Igf1, Ikbkb, Pdpk1, Pik3ca, Pik3cg, Pik3r5, Prkca, Prkcg, Pten, Rps6ka3, Rps6kb1, Rptor, Rragc, Strada, Tsc1, Ulk3, Vegfa	18	5.27E-06
**TGF-β**	miR-141-5p, miR-208a-5p, miR-222-5p, miR-292-3p, miR-299-5p, miR-423-5p, miR-673-5p, miR-1187, miR-1190, miR-487b and miR-671-5p	11	Acvr1c, Acvr2a, Bmp2, Bmp4, Bmp7, Fst, Id4, Inhbb, Nog, Ppp2ca, Rhoa, Rock2, Rps6kb1, Smad1, Smad4, Smurf2, Sp1, Tfdp1, Tgfb2, Tgfbr2, Thbs3, Zfyve9	22	7.18E-06
**VEGF**	miR-141-5p, miR-208a-5p, miR-208b-5p, miR-222-5p, miR-299-5p, miR-673-5p, miR-742-5p, miR-1187, miR-1190 and miR-383	10	Kdr, Map2k1, Mapk11, Nfat5, Nras, Pik3ca, Pik3cg, Pik3r5, Plcg1, Ppp3cb, Prkca, Prkcg, Sh2d2a, Vegfa	14	0.0049
**Apoptosis**	miR-141-5p, miR-208a-5p, miR-208b-5p, miR-222-5p, miR-299-5p, miR-673-5p, miR-742-5p, miR-1187, miR-1190 and miR-671-5p	10	Apaf1, Bcl2, Bid, Birc2, Capn2, Casp6, Casp8, Fasl, Gm9845, Ikbkb, Nfkb1, Pik3ca, Pik3cg, Pik3r5, Ppp3cb, Xiap	16	0.0159
**Up Regulated in MSC vs Cis Comparison**	Overall miRNAs Involved	Number of miRNAs	Overall target genes	Number of Targets	p valor
**TGF-β**	miR-141-5p, miR-291a-3p, miR-292-3p, miR-302b-5p, miR-302d-5p, miR-327, miR-463-3p, miR-568, miR-590-3p, miR-295-5p, miR-503-5p and miR-880-5p	12	Acvr1, Acvr1c, Acvr2a, Bmp5, Bmp8b, Bmpr1a, Dcn, E2f5, Fst, Id2, Inhbb, Inhbe, Lefty1, Lefty2, Ltbp1, Ppp2ca, Rbl1, Rock1, Rock2, Rps6kb1, Smad2, Smad4, Smad6, Smad7, Smurf2, Sp1, Tgfb2, Tgfbr1, Tgfbr2, Thbs1, Zfyve9	31	1.18E-20
**MAPK**	miR-141-5pmiR-208a-3p, miR-291a-3p, miR-292-3pmiR-302d-5p, miR-327, miR-463-3p, miR-568, miR-590-3p, miR-295-5p, miR-302b-5p, miR-503-5p and miR-880-5p	14	Akt3, Atf2, B230120H23Rik, Bdnf, Braf, Cacna1b, Cacna2d1, Cacnb4, Cacng2, Cdc25b, Cdc42, Chuk, Crk, Daxx, Dusp1, Dusp2, Egfr, Elk4, Fgf10, Fgf11, Fgf12, Fgf13, Fgf16, Fgf9, Fgfr2, Flna, Ikbkb, Il1a, Il1r1, Kras, Map3k11, Map3k12, Map3k5, Mapk10, Mapk8, Mecom, Mef2c, Mknk2, Nf1, Ngf, Nlk, Nras, Pak2, Pdgfra, Pla2g4c, Ppp3cb, Ppp3r1, Prkacb, Rac3, Rap1a, Rapgef2, Rasa2, Rasgrf1, Rps6ka1, Rps6ka3, Rps6ka5, Sos1, Sos2, Tab2, Taok1, Tgfb2, Tgfbr1, Tgfbr2, Traf6	64	2.75E-15
**PI3K-Akt**	miR-141-5p, miR-291a-3p, miR-292-3p, miR-302b-5p, miR-302d-5p, miR-327, miR-463-3p, miR-568, miR-590-3p, miR-295-5p, miR-503-5p, miR-541-5p, miR-880-5p and miR-208a-3p	14	Akt3, Atf2, Atf6b, Bcl2, Ccnd2, Ccne1, Ccne2, Cdkn1a, Cdkn1b, Chuk, Col1a1, Col24a1, Col4a2, Col4a4, Col4a6, Col5a1, Col5a2, Creb1, Creb3, Creb3l2, Crtc2, Efna1, Egfr, Eif4b, Fgf10, Fgf11, Fgf12, Fgf13, Fgf16, Fgf9, Fgfr2, Gh, Ghr, Gng2, Gsk3b, Hsp90aa1, Igf1, Ikbkb, Il4ra, Insr, Itga2, Itgav, Itgb1, Itgb8, Kdr, Kras, Lamc1, Lpar4, Mtor, Myb, Ngf, Nras, Pdgfd, Pdgfra, Phlpp2, Pik3ca, Pik3r1, Pkn2, Ppp2ca, Ppp2r2a, Ppp2r2c, Ppp2r3a, Pten, Rbl2, Rps6kb1, Sgk1, Sos1, Sos2, Thbs1, Ywhah	70	1.49E-14
**ErbB**	miR-141-5p, miR-208a-3p, miR-291a-3p, miR-292-3p, miR-302d-5p, miR-327, miR-568, miR-590-3p, miR-302b-5p, miR-503-5p, miR-880-5p and miR-295-5p	12	Akt3, Braf, Cblb, Cdkn1a, Cdkn1b, Crk, Egfr, Erbb4, Ereg, Gab1, Gsk3b, Kras, Mapk10, Mapk8, Mtor, Nck1, Nras, Pak2, Pak7, Pik3ca, Pik3r1, Rps6kb1, Shc4, Sos1, Sos2	25	2.45E-09
**Wnt**	miR-141-5p, miR-208a-3p, miR-291a-3p, miR-292-3p, miR-302d-5p, miR-327, miR-463-3p, miR-568, miR-590-3p, miR-302b-5p, miR-503-5p and miR-295-5p	12	Apc, Btrc, Cacybp, Ccnd2, Csnk1a1, Csnk2a1, Ctbp2, Cxxc4, Dkk1, Fbxw11, Fosl1, Fzd6, Fzd7, Gsk3b, Mapk10, Mapk8, Nfat5, Nlk, Ppp2ca, Ppp3cb, Ppp3r1, Prickle2, Prkacb, Rac3, Rock1, Rock2, Smad2, Smad4, Tbl1x, Wnt16, Wnt3, Wnt3a, Wnt5a, Wnt7a, Wnt8a	35	1.63E-05
**mTOR**	miR-141-5p, miR-291a-3p, miR-292-3p, miR-302d-5p,	10	Akt3, Braf, Cab39, Eif4b, Hif1a, Igf1, Ikbkb, Mtor, Pik3ca, Pik3r1, Pten,	16	6.08E-05

	miR-568, miR-590-3p, miR-302b-5p, miR-503-5p, miR-541-5p and miR-880-5p		Rictor, Rps6ka1, Rps6ka3, Rps6kb1, Strada		
**HIF-1α**	miR-141-5p, miR-291a-3p, miR-292-3p, miR-302d-5p, miR-568, miR-590-3p, miR-302b-5p, miR-503-5p, miR-880-5p and miR-295-5p	10	Akt3, Bcl2, Cdkn1a, Cdkn1b, Cul2, Egfr, Hif1a, Hk3, Igf1, Insr, Mknk2, Mtor, Pdha2, Pfkfb2, Pik3ca, Pik3r1, Rps6kb1, Slc2a1, Stat3, Tfrc	20	0.0291
**Apoptosis**	miR-141-5p, miR-291a-3p, miR-302d-5p, miR-327, miR-463-3p, miR-568, miR-590-3p, miR-302b-5p, miR-503-5p and miR-541-5p	10	Akt3, Apaf1, Bcl2, Chuk, Gm9845, Ikbkb, Il1a, Il1r1, Il1rap, Ngf, Pik3ca, Ppp3cb, Ppp3r1, Prkacb, Prkar2a, Ripk1	17	0.0307
**Down Regulated in MSC vs Cis Comparison**	Overall miRNAs Involved	Number of miRNAs	Overall target genes	Number of Targets	p valor
**ErbB**	miR-693-5p, miR-201-5p and miR-30e-5p	3	Abl2, Braf, Camk2a, Cblb, Crk, Kras, Mapk8, Nck2, Nras, Nrg3, Pik3cd, Sos1	12	0.0008
**VEGF**	miR-543-5p, miR-693-5p, miR-201-5p and miR-30e-5p	4	Kras, Nfat5, Nfatc3, Nras, Pik3cd, Ppp3ca, Ppp3cb, Ppp3r1, Pxn	9	0.0008
**Wnt**	miR-543-5p, miR-693-5p, miR-201-5p and miR-30e-5p	4	Apc, Camk2a, Csnk1a1, Ctnnbip1, Fzd4, Lrp6, Mapk8, Nfat5, Nfatc3, Ppp2r5d, Ppp3ca, Ppp3cb, Ppp3r1, Prickle2, Tbl1xr1, Tcf7l2	16	0.0024
**PI3K-Akt**	miR-377-5p, miR-693-5p, miR-201-5p and miR-30e-5p	4	Bcl2, Bcl2l11, Ccne2, Col4a5, Col5a1, Ddit4, Efna3, Eif4e, Fgf20, Gng10, Ifnar2, Itga8, Itga9, Itgb1, Itgb3, Kit, Kras, Nras, Pik3cd, Ppp2r2a, Ppp2r5d, Prkaa2, Prlr, Sgk3, Sos1, Thbs1, Ywhae, Ywhaz	28	0.0031
**MAPK**	miR-543-5p, miR-693-5p, miR-201-5p and miR-30e-5p	4	Bdnf, Braf, Crk, Fgf20, Il1a, Kras, Map3k12, Map3k5, Mapk8, Nras, Ppp3ca, Ppp3cb, Ppp3r1, Rap1b, Rapgef2, Rasa1, Rasgrp2, Rps6ka2, Rps6ka3, Rps6ka5, Rras2, Sos1, Taok1	23	0.0055
**mTOR**	miR-693-5p, miR-201-5p and miR-30e-5p	3	Braf, Ddit4, Eif4e, Pik3cd, Prkaa2, Rps6ka2, Rps6ka3	7	0.0378
**Apoptosis**	miR-693-5p and miR-30e-5p	2	Bcl2, Casp6, Csf2rb, Il1a, Pik3cd, Ppp3ca, Ppp3cb, Ppp3r1	8	0.0396

Table S3. Compilation of parameters to identification of injury score.

Parameters	0	1	2
Weight loss	< 10 %	10 - 20 %	> 20 %
Posture	Normal	Slightly curved or not erect	Totally curved or not erect
Mobility	Active	Slow or only moves when touched	not moves neither when touched
Hair	Straight	Lightly bristly	Totally bristly and failures
Texture of Feces	Dry	Softened	Pasty
Eyes	Open	Narrowed	Closed
